# Dimensional personality pathology and disordered eating in young adults: measuring the DSM-5 alternative model using the PID-5

**DOI:** 10.3389/fpsyg.2023.1113142

**Published:** 2023-06-26

**Authors:** Tanya Louise Gilmartin, Caroline Gurvich, Joanna F. Dipnall, Gemma Sharp

**Affiliations:** ^1^Department of Neuroscience, Central Clinical School, Faculty of Medicine, Nursing and Health Sciences, Monash University, Melbourne, VIC, Australia; ^2^Monash Alfred Psychiatry Research Centre, Melbourne, VIC, Australia; ^3^School of Public Health and Preventive Medicine, Faculty of Medicine, Nursing and Health Sciences, Monash University, Melbourne, VIC, Australia; ^4^Centre for Innovation in Mental and Physical Health and Clinical Treatment, Faculty of Health, Deakin University, Geelong, VIC, Australia

**Keywords:** disordered eating and eating disorders, personality disorder (PD), PID-5, male eating disorder, personality dimensions

## Abstract

**Introduction:**

The Personality Inventory for DSM-5 (PID-5) is a self-report measure of personality pathology designed to measure pathological personality traits outlined in the DSM-5 alternative model of personality disorders. Within the extensive literature exploring the relationship between personality and disordered eating, there are few that explore the relationship between the PID-5 and disordered eating behaviours in a non-clinical sample of males and females: restrictive eating, binge eating, purging, chewing and spitting, excessive exercising and muscle building.

**Methods:**

An online survey assessed disordered eating, PID-5 traits and general psychopathology and was completed by 394 female and 167 male participants aged 16–30. Simultaneous equations path models were systematically generated for each disordered eating behaviour to identify how the PID-5 scales, body dissatisfaction and age predicted behaviour.

**Results:**

The results indicated that each of the six disordered behaviours were associated with a unique pattern of maladaptive personality traits. The statistical models differed between males and females indicating possible differences in how dimensional personality pathology and disordered eating relate.

**Discussion:**

It was concluded that understanding disordered eating behaviour in the context of personality pathology may assist formulating potentially risky behaviour.

## Introduction

The ongoing engagement in disordered eating behaviours such as fasting, binge eating and compensatory behaviours has been found to impair almost every human physiological process (Mitchell and Crow, 2006) in addition to functional and social impairment (Treasure et al., 2010; Stice et al., 2013). Although disordered eating can occur in the absence of a diagnosed Eating Disorder (ED), engaging in disordered eating has been associated with an increased risk of developing of an ED as well as depression, low self-esteem, anxiety, substance abuse or suicidal behaviours (Ortega-Luyando et al., 2015). Prevalence rates of disordered eating among women range from 0.5%, for self-induced vomiting, to 51.7% for dieting behaviour (Ortega-Luyando et al., 2015).

To understand the reasons why an individual may engage in risky behaviours such as disordered eating, several studies that have investigated the relationship between disordered eating and personality or personality pathology (e.g., Wonderlich et al., 2005; Farstad et al., 2016). Lilenfeld et al. (2006) outlined a number of different models that may explain the relationship between disordered eating behaviour and personality pathology. One model that explains the frequent co-occurrence of disordered eating and personality pathology is labelled the “Spectrum model” which posits that co-occurring disordered eating and personality pathology may be expressions of the same underlying pathology. For example, restrictive eating behaviour may co-occur with rigid, perfectionistic and avoidant behaviours (Díaz-Marsá et al., 2000; Jordan et al., 2008; De Bolle et al., 2011; Farstad et al., 2016; Martinussen et al., 2017) as overt representations of underlying pathology. Similarly, binge eating or purging behaviour has been found to frequently co-occur with impulsive or emotionally dysregulated behaviour (Díaz-Marsá et al., 2000; Bruce and Steiger, 2005; Jordan et al., 2008; Martinussen et al., 2017) and may represent underlying pathology that is distinct from rigid or restrictive patterns, as described above.

### Dimensional approaches to personality and personality pathology

There is a wealth of research focussed on exploring the relationships between personality and EDs and disordered eating. Broadly, research has indicated that eating pathology is associated with traits related to negative affectivity, detachment and conscientiousness (Farstad et al., 2016; Dufresne et al., 2020). However, restrictive eating pathology, such as anorexia nervosa is associated with higher constraint and persistence. In contrast, EDs characterised by binge eating and purging behaviour are more frequently associated with emotion dysregulation and impulsivity. Thus, suggesting diagnostic differences in personality traits (Farstad et al., 2016; Dufresne et al., 2020). A recent systematic review that focussed on the relationship between the Five Factor Model (FFM), a dimensional model of normative personality, concluded that different disordered eating behaviours were found to have unique relationships with personality dimensions. In addition, it was found that exploring facet-level relationships may provide additional insight into understanding the relationships between personality and disordered eating (Gilmartin et al., 2022). In spite of the extensive literature in female populations, there continues to be little information about the relationship between personality and eating disorders and disordered eating among males (Gilmartin et al., 2022, 2023).

**FIGURE 1:**
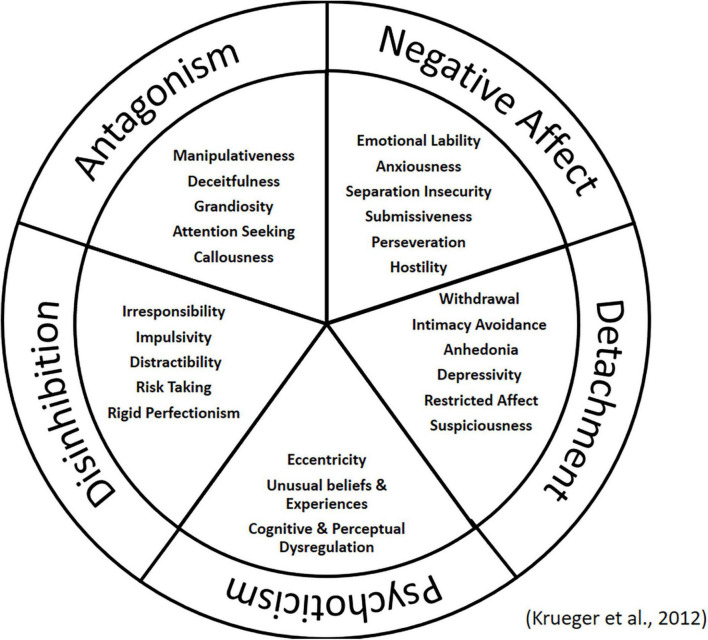
Domains and facets of the PID-5.

While research using the FFM provides us with valuable insight into the relationship between personality and disordered eating, it is important to note that as a model of normal personality, there are both adaptive and maladaptive variants of each domain and facet (Widiger and Costa, 2012). What is missing is insight into the pathological dimensions and how these relate to eating pathology. With the publication of the fifth edition of the Diagnostic and Statistical Manual of Mental Disorders (DSM-5), an alternative model to the categorical approach to personality disorder diagnosis was introduced as an area for further research. The DSM-5 Alternative Model (AMPD) considers personality disorders as “maladaptive variants of personality traits that emerge imperceptibly into normality and into one another” (American Psychiatric Association [APA], 2013, p. 646). The inclusion of pathological trait domains was designed to assign a diagnosis that fits the specific patient (Skodol, 2012). The Personality Inventory for DSM-5 (PID-5) was designed as a measure of the 25 maladaptive trait facets and five higher-order domains included in the model (see Figure 1; Krueger et al., 2012; Fowler et al., 2017). Although the AMPD model also consists of a measure of severity of personality dysfunction (American Psychiatric Association [APA], 2013). This was not the focus of our research.

While there is robust research literature exploring co-occurring personality disorders and eating disorders (Farstad et al., 2016; Dufresne et al., 2020) the exploration of disordered eating and the PID-5 is in it’s infancy. Solomon-Krakus et al. (2019) explored theoretical models as predictors of restrictive eating, binge eating and compensatory behaviour among 570 non-clinical adults. Higher scores on the PID-5 Rigid Perfectionism scale were associated with restriction only when males and female data was considered together. The researchers suggested that this may reflect either the study’s non-clinical nature or a lack of statistical power (Solomon-Krakus et al., 2019). In another study that explored personality among women of a higher weight with and without binge eating disorder, higher scores on the Anhedonia, Emotional Lability, Impulsivity, and Depressivity subscales were associated with features of binge eating disorder (Aloi et al., 2020). Although limited, research focussed on the relationship between the PID-5 and disordered eating behaviours indicate consistencies with the broader research literature that has indicated a positive association between restrictive eating and rigidity, perfectionism and social avoidance (De Bolle et al., 2011; Reas et al., 2013; Farstad et al., 2016). Likewise, the results with the PID-5 and binge eating behaviour are consistent with findings that features such as impulsivity and low mood are associated with binge eating behaviour (Zanarini et al., 2010; De Bolle et al., 2011; Reas et al., 2013; Farstad et al., 2016).

### The present study

Despite the extensive research literature on the relationship between diagnosed EDs and personality or personality pathology, the relationship between disordered eating and the AMPD and the associated PID-5 remains more preliminary. Therefore, the overall aim of the present study is to extend on the limited literature to date by exploring predictive models of six disordered eating behaviours among a sample of non-clinical males and females using PID-5 measure of personality pathology. The PID-5 was selected as the measure of personality pathology for use in this study due to its design as a measurement of DSM-5 personality dimensions (Krueger et al., 2012; Fowler et al., 2017) and to add to the limited existing research. For the current study, it was decided that the focus would be disordered eating behaviours rather than general eating pathology or ED diagnostic criteria. This decision was made based on findings that have indicated high levels of disordered eating behaviour in non-clinical populations (Ortega-Luyando et al., 2015), and the growing body of literature indicating that personality traits tend to be more reliably related to ED symptoms rather than ED diagnoses (Dufresne et al., 2020). The disordered eating behaviours in focus in the current study were restrictive eating, binge eating, purging, excessive exercise and muscle building, based on research that has differentiated these behaviours as core eating disorder symptoms (Forbush et al., 2013). Chewing and spitting has been found to be increasing in prevalence among Australian adolescents (Aouad et al., 2021) and exists among samples of Australian adults (Aouad et al., 2018), thus, it was included as a sixth behaviour of focus in this study.

Studies exploring gender differences in disordered eating behaviour have suggested that disordered eating has been increasing at a faster rate among males compared to females (Gorrell and Murray, 2019). Although females tend to restrict their diet or purge more frequently than males, males tend to be equally as likely to binge eat (Mitchison et al., 2014), and may engage in behaviours intended to achieve a muscular physique (Gorrell and Murray, 2019). Gender differences have been identified in the relationship between normative personality and disordered eating, in that different personality traits have been found to be associated with the same disordered eating behaviour (Gilmartin et al., 2022). However, within the limited research literature exploring disordered eating and the PID-5, no differences have thus far been identified (Solomon-Krakus et al., 2019). Therefore, we examined the relationship between personality and disordered eating separately between males and females so that we could explore the relationships unique to males and females. We also used a measure of disordered eating that has been designed to detect disordered eating behaviours that may be more relevant for males such as exercise and building muscle (Forbush et al., 2014) in addition to restrictive eating, binge eating, purging and chewing and spitting.

Given the exploratory nature of the current study, key variables were included in our analyses. Specifically, personality has been found to predict body image dissatisfaction (Allen and Walter, 2016; Allen and Robson, 2020). In addition, body image dissatisfaction is considered a core feature associated with the development and maintenance of an ED (Fairburn, 2008; Espinoza et al., 2010; Liechty and Lee, 2013; Gitimu et al., 2016), and has been found to predict eating behaviour (DuBois et al., 2017; Devrim et al., 2018). Body image concerns have also been previously found to mediate the relationship between personality and measures of general mental health (Allen and Celestino, 2020). It was therefore considered a possibility that body image dissatisfaction may play a mediating role in the relationship between personality and disordered eating. Second, eating disorders and disordered eating have been found to have an adolescent age of onset (Volpe et al., 2016). In addition, past research has indicated that chewing and spitting is more likely to occur in younger individuals (Aouad et al., 2018). Therefore, age was included as a predictor variable in statistical models.

There were three broad aims of the current study:

• Aim 1: To explore the relationship between the PID-5 and six disordered eating behaviours (restrictive eating, binge eating, purging, chewing and spitting, excessive exercise, and muscle building). Based on limited past research it was expected that restrictive eating behaviour would be associated with increased PID-5 rigid perfectionism, and that binge eating behaviour would be associated with increased PID-5 anhedonia, emotional lability, impulsivity, anxiousness, and depressivity.
• Aim 2: To explore the relationship between PID-5 personality dimensions and disordered eating separately for males and females to identify models of personality that are specific for both genders.
• Aim 3: To explore how body image dissatisfaction may mediate the relationship between the PID-5 and disordered eating, after controlling for the potential confounding influence of age.

## Materials and methods

### Participants

All procedures were approved by (Monash University’s) institutional Ethics Committee prior to study commencement. The current research was conducted as part of a wider study exploring the relationship between personality and disordered eating among young Australians. Another paper using this data set reporting on the relationship between disordered eating and the FFM also includes a summary flowchart of participant engagement in the study Table 1 summarises the demographic details of the sample. The current sample has been described in more detail elsewhere, and Table 1 has been previously published (Gilmartin et al., 2023). The final sample consisted of 571 individuals, aged 16–30 (*M* = 22.15, SD = 3.84), including 167 Males (29.4%, *M* = 21.76, SD = 3.62), 394 females (69.00%, *M* = 22.31, SD = 3.94), and 10 individuals who identified as another gender (1.75%, *M* = 21.8, SD = 3.05). The sample was mainly White (70%) and/or had a Year 12 (final year of high school) or equivalent education (45%).

**TABLE 1:** Demographic information of the sample.

Demographics	Males *N* = 167	Females *N* = 394	Gender diverse *N* = 10	Total *N* = 571
**Ethnicity**				
White	108 (64.67%)	280 (71.07%)	7 (70%)	395 (69.18%)
Asian	49 (29.34%)	84 (21.32)	2 (20%)	135 (23.64%)
Mixed race	6 (3.39%)	20 (5.08)	–	26 (15.20%)
South Asian	3 (1.80%)	1 (0.25%)	1 (10%)	5 (0.88%)
African	0	3 (0.76%)	–	3 (0.53%)
Aboriginal/Torres Strait Islander	1 (0.60%)	1 (0.25%)	–	2 (0.35%)
Other	0	5 (1.27%)	–	5 (0.88%)
**Highest year education^†^**				
Year 10 (or equivalent)	5 (2.99%)	6 (1.52%)	2 (20%)	13 (2.28%)
Year 11 (or equivalent)	4 (2.40%)	14 (3.55%)	–	18 (3.15%)
Year 12 (or equivalent)	82 (49.10%)	171 (43.40%)	5 (50%)	258 (45.18%)
Diploma	14 (8.38%)	21 (5.33%)	–	35 (6.13%)
Bachelor’s degree	48 (28.74%)	133 (33.76%)	1 (10%)	182 (31.87%)
Postgraduate degree	14 (8.38%)	49 (12.44%)	2 (20%)	65 (11.38%)
**Sexual orientation^∓^**				
Heterosexual	136 (81.44%)	259 (65.74%)	1 (10%)	396 (69.35%)
Bisexual	11 (6.59%)	87 (22.08%)	4 (40%)	102 (17.86%)
Lesbian or gay	14 (8.38%)	10 (2.54%)	2 (20%)	26 (4.55%)
Something else	4 (2.40%)	13 (3.30%)	3 (30%)	20 (3.50%)
Unsure	2 (1.20%)	25 (6.35%)	–	27 (4.73%)
**Self-report clinical diagnoses**				
Mood disorder	22 (13.17%)	118 (29.95%)	5 (50%)	145 (25.39%)
Anxiety disorder	19 (11.38%)	129 (32.74%)	7 (70%)	155 (27.15%)
Anorexia nervosa	2 (1.20%)	32 (8.12%)	–	34 (5.95%)
Bulimia nervosa	1 (0.60%)	11 (2.79%)	–	12 (2.10%)
Binge eating disorder	–	2 (0.51%)	–	2 (0.35%)
ARFID	–	1 (0.25%)	–	1 (0.18%)
Other eating disorder	1 (0.60%)	11 (2.79%)	–	12 (2.10%)
Borderline personality disorder	2 (1.20%)	28 (7.10%)	1 (10%)	31 (5.43%)
OCD	2 (1.20%)	14 (3.55%)	–	16 (2.80%)
ADHD/ADD	1 (0.60%)	9 (2.28%)	1 (10%)	11 (1.93%)
Autism	1 (0.60%)	2 (0.51%)	–	3 (0.53%)
None specified	130 (77.38%)	203 (51.52%)	3 (30%)	336 (58.84%)

Participants were able to self-report as many clinical diagnoses as relevant. ARFID, avoidant/restrictive food intake disorder; OCD, obsessive compulsive disorder; ADHD/ADD, attention-deficit hyperactivity disorder/attention-deficit disorder.

^†^Year 12 is the final year of high school in Australia.

^∓^A chi-square test of independence was performed to examine differences between males and females in demographic variables. Only the following differences were found: males were more likely to be gay, while females were more likely to be bisexual or unsure X^2^ (4, *N* = 561) = 36.37, *p* < 0.001.

### Measures

The survey completed by participants measured DSM-5 personality traits and eating behaviour. All measures used in the current study have been previously and appropriately used with participants as young as 16 years old (Ro et al., 2012; De Clercq et al., 2014; Shaw et al., 2017; Forbush et al., 2018; Christian et al., 2020; See et al., 2020).

#### Personality pathology

The PID-5 Short-Form (PID-5-SF; Maples et al., 2015) was used in the current study as a measure of personality pathology. The PID-5 SF is a 100 item measure of DSM-5 personality disorder domains, measuring the five domains (negative affectivity, detachment, antagonism, disinhibition, and psychoticism), and 25 facets featured within the PID-5. The scale asks participants if items reflect how they may describe themselves (e.g., “I am easily angered,” “I’m always worrying about something”) and are scored on a four-point Likert scale ranging from 0 (very false or often false) to 3 (very true or often true). Higher scores indicate higher pathology. The four items measuring each facet are summed and the Mean computed for facet scores. The short form has been found to be highly correlated with the original scale (Maples et al., 2015). In the current sample, majority of the subscales demonstrated adequate to strong internal consistency (α = 0.71–0.91) with the exception of Irresponsibility which demonstrated questionable internal consistency (α = 0.61). It was decided to retain the scale to maintain a consistent approach to analysing each of the PID-5 domains.

#### Eating behaviour

The Eating Pathology Symptoms Inventory (EPSI; Forbush et al., 2013) was used to assess disordered eating behaviours. It is a 45-item scale that is scored on a five-point Likert scale ranging from 0 (Never) to 4 (Very often). The scale is divided into eight subscales, with each of the subscale items summed together to obtain a total score. Higher scores indicate higher levels of disordered eating. The measure includes the Body Dissatisfaction subscale (e.g., “I did not like how clothes fit the shape of my body”) that provides a measure of concerns with body shape. Five subscales were selected as measures of disorder eating in the current study. Restriction assessed reduced food consumption, the Binge eating scale measures the ingestion of large amounts of food. The Purging subscale is designed to assess self-induced vomiting, laxative use, diuretic use, and diet pill use. Excessive Exercise measures compulsive or intense exercise and Muscle Building assesses efforts to build muscle and the engagement in supplement use. In order to assess chewing and spitting behaviour, an additional item was added (“I spat out food after chewing to avoid putting on weight”). This item was based on items used in surveys administered by Aouad et al. (2021) and worded to remain consistent with the other items in the EPSI. Two EPSI subscales were omitted from the study. The Cognitive Restraint and Negative Attitudes Toward Obesity were considered to be more focussed on internal processes rather than behaviour so were not consistent with the goals of the current study. The internal consistency in the current study was found to range from good to strong (α = 0.79–0.90). The EPSI has previously been found to have good test–retest reliability for all scales for men and women together, in addition to being invariant across gender (Forbush et al., 2013), and for most scales when genders were considered separately (Forbush et al., 2019).

The Eating Disorder Examination Questionnaire-Short version (EDE-QS) has been developed as a 12-item version of the full scale measuring general eating disorder psychopathology (Gideon et al., 2016). Participants select on how many days they engaged in particular behaviours (e.g., “Have you had a strong desire to lose weight?” “Have you had a sense of having lost control over your eating?”) with items are scored on a four-point Likert scale ranging from 0 (0 days) to 3 (6–7 days). The EDE-QS was included as an assessment of overall eating pathology in contrast to the EPSI which assesses specific disordered eating behaviours. The scale has been found to have strong internal consistency, with a Cronbach’s alpha coefficient of α = 0.91 in the current sample. The EDE-Q has been found to be appropriate for use with both males and females (Rand-Giovannetti et al., 2020).

#### Negative emotional states

The Depression Anxiety Stress Scale-21 item version (DASS-21) was used to measure negative mood states. The DASS-21 is a shortened version of the original 42-item DASS. The Depression subscale is designed to assess low positive affect (e.g., “I couldn’t seem to experience any positive feeling at all”). The Anxiety subscale measures physical hyperarousal (e.g., “I felt I was close to panic”) and the Stress subscale measures tension or irritability (e.g., “I found it difficult to relax”; Antony et al., 1998). Scoring for the DASS-21 is based on a 4-point Likert scale ranging from 0 (Did not apply to me at all/Never) to 3 (Applied to me very much or most of the time/Almost Always; Henry and Crawford, 2005). Research has replicated the three-factor structure of the DASS and DASS-21 (Antony et al., 1998; Henry and Crawford, 2005) and the DASS-21 demonstrated good internal consistency in the current sample (α = 0.84–0.91).

### Procedures

We used an online survey consisting of demographic questions and psychometric measures, described above, to collect data for the current study. The survey was advertised using social media posts and electronic newsletters associated with ED and personality disorder organisations, university pages, community and sport notice boards, and pages associated with interest groups such as fitness, trades and food. Prospective participants were provided a brief outline of the study and what was involved in participation. Participants acknowledged they had read the information and provided consent via checking a box, before beginning the survey. The survey consisted of questions to collect demographic information and psychometric measures. Participants were informed that they could enter a draw to win a voucher worth $35 USD.

### Statistical analysis

**FIGURE 2:**
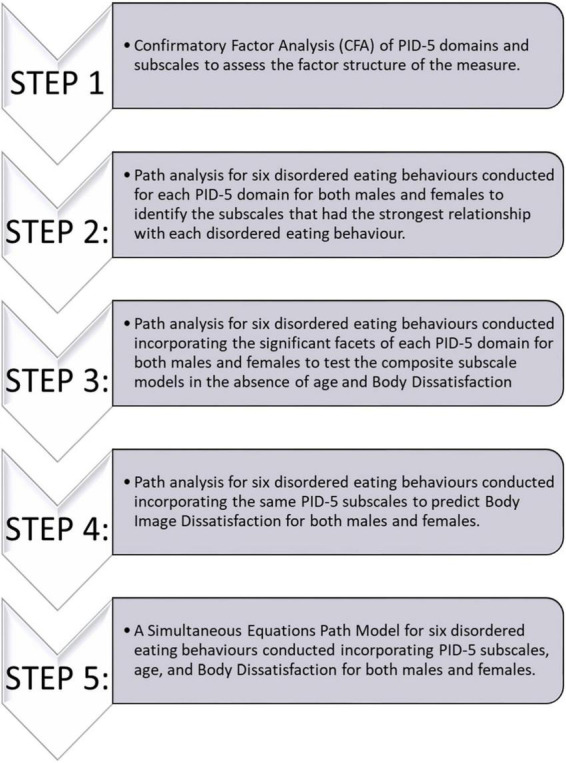
Data analysis flowchart.

The statistical procedure used in the current study has been outlined in Figure 2. Two participants had missed items throughout the study and were excluded from the path analyses. First, a Confirmatory Factor Analysis (CFA) was performed to verify the relationship between observed variables and underlying latent trait domains explored in the PID-5, and to ensure that the latent structure of the PIDF-5 existed within our data (see Supplementary material). Then a theoretical path model for the facets within each PID-5 trait domain explored how the facets within a domain were associated with eating behaviour. A less stringent alpha value was implemented (*p* < 0.1) to account for potential suppressor effects of other scales within the same domain for steps two and three. In order to allow for continuity of analysis across genders, the sexual orientation was excluded as a control variable due to the large difference in proportions between females and males who did not identify as heterosexual. A sensitivity analysis was performed, where models including sexual orientation as a control were compared to those without, confirmed this decision as very few differences were found between the models.

The data analysis process resulted in 12 Simultaneous Equations (see section “Path models”). Chi-square was reported to assess the absolute fit of each model. In addition, the fit of each model was evaluated using the Root Mean Squared Error of Approximation (RMSEA), the comparative fit index (CFI), and the Tucker Lewis Index (TLI). RMSEA values that approximated 0.06 or lower, and CFI and TLI values that approximated 0.95 or higher, indicated better fitting models (Hu and Bentler, 1999). Statistical *post hoc* power calculations were conducted for the models based on RMSEA using the R statistical software (Preacher and Coffman, 2006).

The indirect path analysis was calculated using 1,000 bootstrapped samples, and bias-corrected 95% confidence intervals. The direct, indirect and total effects have been reported in this paper, along with the bootstrapped 95% confidence intervals. All statistical calculations were implemented using SPSS version 27 (IBM Corp, 2020) and AMOS version 26 (Arbuckle, 2019).

## Results

Table 2 displays the means and standard deviations for age, EPSI, PID-5-SF, DASS-21, and EDE-QS by gender, and comparative *t*-tests between males and females. The Independent Samples *t*-tests reported in this study have also been reported elsewhere (Gilmartin et al., 2023). A minority of participants (1.75%) identified as gender diverse and due to the small sample size they were not included in subsequent analyses. The means and standard deviations for each measure for males, females and gender-diverse participants have been included in the Supplementary material. The scores obtained on the EPSI in the current study appeared to be lower compared to normative data in a college sample published by Forbush et al. (2014), while scores obtained on the DASS-21 appeared substantially higher than normative data published by Henry and Crawford (2005) or Crawford et al. (2020); see Supplementary material. There was no comparative normative data for the PID-5 SF, however, the scores obtained in the current sample were found to be within one standard deviation of the mean for each subscale when compared to Miller et al. (2022) PID-5 (full scale) norms. The exceptions to this were for females, who scored higher on the Negative Affectivity domain and the Anxiousness and Grandiosity facets. It is unclear if the difference was observed as a result of the reduced number of items on the PID-5 SF compared to the PID-5, because the female data from this study was compared to data that was a combination of male and female in Miller et al. (2022)’s paper, or reflective of the current sample.

**TABLE 2:** Model fit indices for each path model.

Model	Males				Females			
	χ^2^(*df*)	CFI	TLI	RMSEA (90% CI)	χ^2^ (*df*)	CFI	TLI	RMSEA (90% CI)
Restrictive eating	17.74 (14)^∧^	0.976	0.937	0.040 (0.000–0.090)	16.84 (22)†	0.997	0.988	0.024 (0.000–0.051)
Binge eating	48.74*(24)^†^	0.939	0.861	0.079 (0.046–0.111)	12.03 (17)^†^	1.000	1.015	0.000 (0.000–0.030)
Purging	22.20 (13)^∧^	0.980	0.929	0.065 (0.000–0.110)	31.08 (23)^†^	0.994	0.980	0.031 (0.000–0.055)
Chew and spit	4.20 (2)^∧^	0.994	0.952	0.081 (0.000–0.192)	21.21 (20)^†^	0.999	0.996	0.012 (0.000–0.046)
Excessive exercise	13.46 (16)^∧^	1.000	1.024	0.000 (0.000–0.060)	21.04 (12)^†^	0.991	0.964	0.044 (0.001–0.074)
Muscle building	27.44 (22)^†^	0.981	0.952	0.039 (0.000–0.079)	11.22 (7)^†^	0.988	0.965	0.039 (0.000–0.080)

*p < 0.05. Power: ^∧^Low < 0.8. ^†^Acceptable ≥ 0.8.

The correlation matrix (see Supplementary material) indicated strong positive correlations between the EPSI Body Dissatisfaction scale and the EDE-QS (Males: *r* = 0.69, *p* < 0.001; Females: *r* = 0.73, *p* < 0.001) and between the PID-5 Anhedonia (Males: *r* = 0.65, *p* < 0.001; Females: *r* = 0.78, *p* < 0.001) or Depressivity (Males: *r* = 0.70, *p* < 0.001; Females: *r* = 0.78, *p* < 0.001) subscales and the DASS-21 Depression scale. The strong correlation coefficients are indicative of multicollinearity which would cause instability in the regression coefficients. Therefore, the EDE-QS and DASS-21 scores were not included in subsequent analyses.

### Path models

As demonstrated in Figure 2, the PID-5 subscales that were the strongest predictors within each of the five domains for each disordered eating behaviour were added to a composite path model to identify the facets that were the strongest predictors of disordered eating behaviour. Age was added as a predictor to each model, and the EPSI Body Dissatisfaction scale was added as a mediator in each model. The results of analyses from Steps 1 to 4 have been included in the Supplementary material. The model fit indices for each model have been displayed in Table 2. The models for males were underpowered and as a result these were not an optimal fit of the data. The female models were found to be a good fit of the data.

#### Disordered eating behaviour in males

**FIGURE 3:**
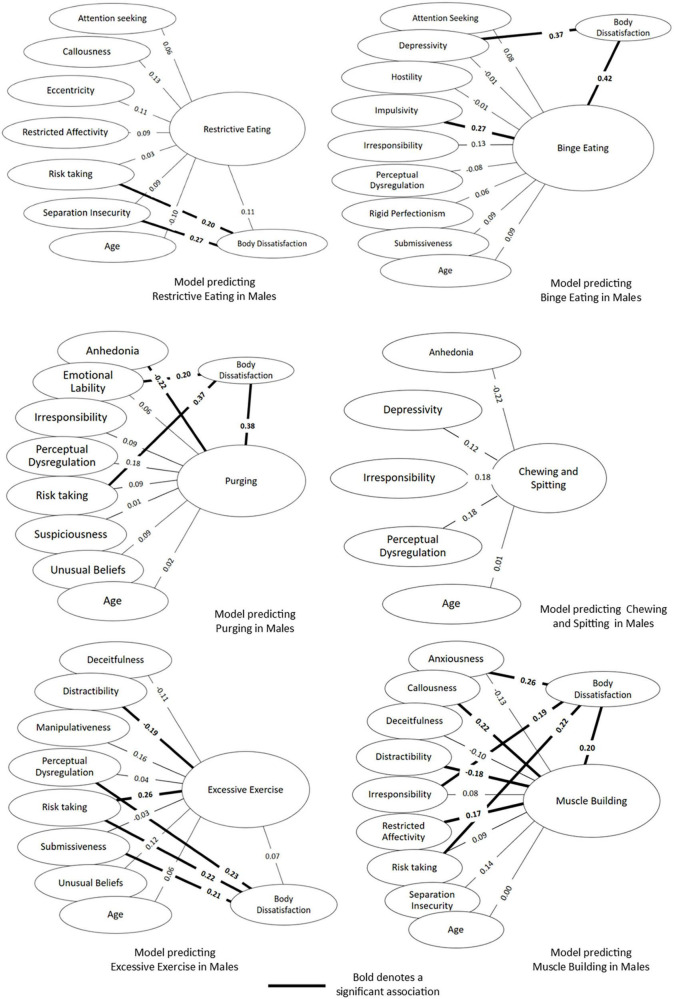
Simultaneous equations path models predicting six disordered eating behaviours among males.

The results for the six path models predicting disordered eating in males specifically have been displayed as Figure 3. The Direct, Indirect and Total effects for each model are displayed in Table 3. For Restrictive Eating and Chewing and Spitting, no PID-5 scales or EPSI Body Dissatisfaction were found to be significant predictors. To note, at step 4, none of the PID-5 facets were found to significantly predict Body Dissatisfaction in the Chewing and Spitting model, so EPSI Body Dissatisfaction was not included in the final path model. For the Binge eating model, a tendency to act without planning was the only PID-5 scale to directly predict Binge Eating among males in a positive direction, while a tendency to have a low mood and be pessimistic was found to Indirectly predict binge eating through Body Dissatisfaction. A lack of enjoyment in life and Body Dissatisfaction were the only direct predictors of purging behaviours. An instability of emotions and tendency to disregard personal risk were both found to indirectly predict Purging behaviour through Body Dissatisfaction. When looking at the model for Excessive Exercise, an ability to remain focussed in addition to a tendency to engage in behaviours regardless of potential risks were found to directly predict excessive exercise among males. Considering the Muscle Building path model in Figure 3, a lack of concern for the feelings of others, goal-focussed behaviour, a constricted emotional experience and body dissatisfaction directly predicted muscle building behaviour in Males. Increased fearfulness, carelessness and a lack of concern for consequences were positively associated with increased body dissatisfaction, which, in turn, led to increased muscle building behaviour.

**TABLE 3:** Direct, indirect and total effects for each path model for eating behaviours in males.

PID-5 subscale	Total effects		Direct effects		Indirect effects	
	*b*	95% CI	*b*	95% CI	*b*	95% CI
**Restrictive eating**						
Risk taking	0.05	−0.111, 0.217	0.03	-0.140, 0.211	0.02	−0.006, 0.077
Separation insecurity	0.12	−0.048, 0.281	0.09	-0.085, 0.254	0.03	−0.008, 0.085
**Binge eating**						
Depressivity	0.15	−0.038, 0.333	-0.01	−0.182, 0.163	0.15*	0.080, 0.254
**Purging**						
Emotional lability	0.20*	0.056, 0.326	0.06	−0.099, 0.199	0.14*	0.074, 0.232
Risk taking	0.16	−0.14, 0.300	0.09	−0.068, 0.217	0.07*	0.018, 0.141
**Excessive exercise**						
Perceptual dysregulation	-0.02	−0.259, 0.214	-0.04	−0.263, 0.192	0.02	-0.018, 0.074
Risk taking	0.28*	0.114, 0.417	0.26*	0.091, 0.402	0.02	−0.016, 0.070
Submissiveness	-0.02	−0.178, 0.145	-0.03	−0.201, 0.134	0.02	−0.015, 0.065
**Muscle building**						
Anxiousness	-0.08	−0.232, 0.114	-0.13	−0.291, 0.060	0.05*	0.005, 0.133
Irresponsibility	0.12	−0.036, 0.281	0.08	−0.066, 0.248	0.04*	0.006, 0.097
Risk taking	0.14	−0.014, 0.310	0.09	−0.069, 0.276	0.05*	0.007, 0.119

*p < 0.05. b = Standardised coefficient. Indirect effects = mediation. *CI* = Bias-corrected bootstrapped confidence interval (1000 samples).

#### Disordered eating behaviour in females

**FIGURE 4:**
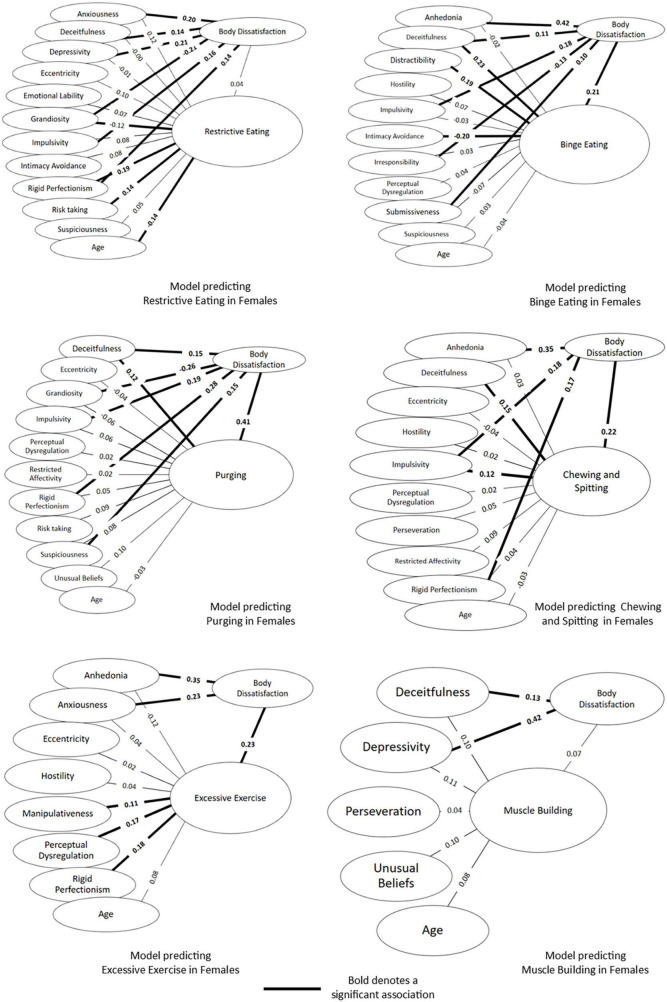
Simultaneous equations path models predicting six disordered eating behaviours among females.

The results for the six path models predicting disordered eating in females have been displayed as Figure 4. The direct, indirect and total effects for each model are displayed in Table 4. Restrictive eating in females was predicted by a tendency to be rigidly focussed on avoiding fault, in addition to being younger, possessing a tendency to engage in behaviours regardless of risk and not endorse feelings of superiority. When considering the Binge Eating path model, a tendency to be dishonest, avoidance of relationships and a difficulty concentrating was predictive of Binge Eating. Additionally, a lack of enjoyment, acting without thinking and a tendency to go against one’s own needs to connect with others were found to predict Binge Eating indirectly through Body Dissatisfaction. For Purging, dishonesty and a dissatisfaction with appearance were the only direct predictors, while a sense of urgency, focus on correctness and low grandiosity were found to predict Purging indirectly through body dissatisfaction. The Chewing and Spitting model showed that body dissatisfaction along with dishonesty and acting without thinking were significant predictors. A need for correctness was found to predict Chewing and Spitting indirectly through Body Dissatisfaction. For Excessive Exercise, in addition to high Body Dissatisfaction, a need for correctness, odd and unusual thought processes and manipulativeness were predictive of Excessive Exercise behaviour. A lack of enjoyment and tendency to worry were both found to indirectly predict exercise through Body Dissatisfaction. No variables were found to directly or indirectly predict Muscle Building behaviour, however, there were significant total effects for depressivity, indicating increased scores on depressivity were associated with increased Muscle Building behaviour when body dissatisfaction was taken into account.

**TABLE 4:** Direct, indirect and total effects for each path model for eating behaviours in females.

PID-5 subscale	Total effects		Direct effects		Indirect effects	
	*b*	95% CI	*b*	95% CI	*b*	95% CI
**Restrictive eating**						
Anxiousness	0.12*	0.008, 0.236	0.12*	0.003, 0.228	0.01	−0.012, 0.036
Deceitfulness	0.00	−0.039, 0.124	0.00	−0.147, 0.122	0.01	−0.008, 0.025
Depressivity	-0.00	−0.152, 0.140	-0.01	−0.168, 0.127	0.01	−0.015, 0.034
Grandiosity	0.13*	−0.224, −0.032	-0.12*	−0.218, −0.027	-0.01	−0.038, 0.013
Impulsivity	0.09	−0.035, 0.213	0.08	−0.044, 0.209	0.01	−0.009, 0.031
Rigid perfectionism	0.20*	0.094, 0.301	0.19*	0.083, 0.298	0.01	−0.008, 0.027
**Binge eating**						
Anhedonia	0.07	−0.058, 0.206	-0.02	−0.164, 0.118	0.09*	0.037, 0.142
Deceitfulness	0.26*	0.136, 0.366	0.23*	0.116, 0.343	0.02*	0.003, 0.053
Impulsivity	0.03	−0.122, 0.157	-0.01	−0.159, 0.119	0.04*	0.013, 0.072
Irresponsibility	0.00	−0.121, 0.135	0.03	−0.089, 0.157	-0.03	−0.067, 0.004
Submissiveness	0.09	−0.010, 0.176	0.07	−0.028, 0.152	0.02*	0.003, 0.049
**Purging**						
Deceitfulness	0.18*	0.043, 0.321	0.12	−0.008, 0.254	0.06*	0.022, 0.109
Grandiosity	-0.16*	−0.256, −0.076	-0.06	−0.143, 0.037	-0.11*	−0.160, −0.062
Impulsivity	0.14*	0.020, 0.259	0.06	−0.055, 0.173	0.08*	0.038, 0.124
Rigid perfectionism	0.17*	0.076, 0.268	0.05	−0.038, 0.137	0.12*	0.072, 0.164
Suspiciousness	0.14*	0.014, 0.283	0.08	−0.037, 0.207	0.06	0.021, 0.108
**Chewing and Spitting**						
Anhedonia	0.11	−0.021, 0.250	0.03	−0.122,0.170	0.08	0.041, 0.127
Impulsivity	0.16*	0.037, 0.270	0.12	−0.005, 0.228	0.04*	0.019, 0.075
Rigid perfectionism	0.08	−0.038, 0.202	0.04	−0.073, 0.156	0.04*	0.015, 0.080
**Excessive exercise**						
Anhedonia	-0.03	−0.169, 0.104	-0.12	−0.259, 0.027	0.08*	0.041, 0.140
Anxiousness	0.09	−0.025, 0.208	0.04	−0.074, 0.160	0.05*	0.022, 0.092
**Muscle building**						
Deceitfulness	0.11	−0.024, 0.216	0.10	−0.031, 0.211	0.01	−0.004, 0.029
Depressivity	0.14*	0.001, 0.266	0.11	−0.039, 0.247	0.03	−0.015, 0.074

*p < 0.05. b = Standardised coefficient. Indirect effects = mediation. *CI* = Bias-corrected bootstrapped confidence interval (1000 samples).

## Discussion

We explored three aims to further understand the relationship between disordered eating and the PID-5 for males and females separately, and the influence of body dissatisfaction. The results demonstrate that among young people in Australia, both males and females engage in a range of disordered eating behaviours. When considering the aims of the study, there were differences in statistical models between males and females for each studied disordered eating behaviour. Body image dissatisfaction was found to play a unique role in each model. Our study provides a unique contribution and extension to the previous literature by exploring the relationship between the AMPD traits and a number of disordered eating behaviours that have not been previously explored together. Our findings highlight the importance of understanding disordered eating behaviours in the context of dimensional personality pathology. The key findings of the current study in the context of the first aim of this study are explored below.

### Disordered eating behaviours among males

The results of the study revealed different predictive models for each disordered eating behaviour under investigation. Body dissatisfaction was associated with binge eating, purging and muscle building among males. One key finding was that a tendency to act without thinking was associated with binge eating, which is consistent with the current literature which has linked impulsivity to binge eating both using the PID-5 (Solomon-Krakus et al., 2019) and more broadly (Kittel et al., 2017; Magel and von Ranson, 2021; Gilmartin et al., 2022, 2023). Importantly, we extended on the Aloi et al. (2020) female sample, by finding that PID-5 depression is also associated with binge eating in males, and that the relationship is mediated by body dissatisfaction.

The spectrum model as outlined by Lilenfeld et al. (2006) posits that disordered eating and personality pathology may tend to co-occur because they represent maladaptive expressions of one underlying condition. A parallel can be drawn with psychodynamic conceptualisations of maladaptive behaviour as reflecting underlying personal factors (Westen et al., 2006). Within this framework, our research highlights four novel examples of how these theoretical constructs may be translated into behaviour. First, a tendency to be overly goal-focussed was found to be associated with both excessive exercise and muscle building. It may be implied that excessive exercise or muscle building behaviour may represent maladaptive variations of low PID-5 Distractibility scores. While this is a new finding among males, it may provide an extension of the existing literature that has found that self-discipline is a predictor of excessive exercise among females (Dalle Grave et al., 2008).

Second, a tendency to act in a manner that increases personal risk was associated with purging, excessive exercise and muscle building, although the relationship was mediated by body dissatisfaction within the purging and muscle building models. Taken together, the current results may indicate a willingness to disregard the potential risk of disordered eating behaviours (Vandereycken and Van Humbeeck, 2008) in favour of focussing on goals which may serve to relieve body image distress (Donovan et al., 2020).

Thirdly, in parallel with Gilmartin et al. (2022), we found that a lack of concern for others and constricted emotional experience was associated with muscle building in males. As an extension, a lack of responsibility for the needs of others was found to be indirectly associated with muscle building through body dissatisfaction in this study. When considering these traits together, the current results link social insensitivity to exercise behaviour among females (Dalle Grave et al., 2008).

Finally, as an important extension on the current research literature (Weinstein et al., 2015; Brown et al., 2020; Legg and Turner, 2021), our models suggest that purging behaviour may represent an expression of emotional instability, and muscle building an expression of anxiety when an individual experiences dissatisfaction with their appearance. However, further research is required to gain an in-depth understanding of this relationship.

None of the personality variables or body dissatisfaction included in the final models were found to be associated with restrictive eating or chewing and spitting among males. Previous research has also not found any relationships between restrictive eating and the PID-5 (Solomon-Krakus et al., 2019) or body dissatisfaction (Gilmartin et al., 2023) in males. In the same sample, normative personality traits were found to be predictive of disordered eating among males (Gilmartin et al., 2023), suggesting that measures of normative and pathological personality differ in their capabilities of predicting disordered eating in males. This represents an important platform for future research.

### Disordered eating behaviours among females

Consistent with the statistical models generated for males in this study in addition to past research (Gilmartin et al., 2023), there was considerable variability among the statistical models generated for female eating behaviours. We will outline each of the key contributions for our study separately. Our findings were inconsistent with previous studies that have consistently linked impulsivity to binge eating or purging behaviour (Solomon-Krakus et al., 2019; Aloi et al., 2020; Legg and Turner, 2021; Magel and von Ranson, 2021; Gilmartin et al., 2022, 2023). Our findings extend on past research by revealing a direct relationship between impulsivity and chewing and spitting among females. There is a paucity of past research exploring chewing and spitting behaviour, and although the current findings are novel, it does provide support for and add to research that has indicated that chewing and spitting behaviour may represent a maladaptive coping strategy through impulsive reactions to uncomfortable stimuli in adolescents (Aouad et al., 2020).

The hypothesis that there would be a relationship between PID-5 Perfectionism and restrictive eating was supported in the current study, along with past research suggesting the same (Solomon-Krakus et al., 2019). In the context of the broader research literature, the current results add further support for the association between perfectionism and restrictive eating (Forbush et al., 2007; Donovan et al., 2014; Dufresne et al., 2020) and compulsive exercise (Flett and Hewitt, 2005; Goodwin et al., 2011; Çakın et al., 2021), and provides an important addition to past research by indicating that body dissatisfaction mediates the relationship between perfectionism and purging (Forbush et al., 2007) or chewing and spitting.

Interpersonal difficulties have previously been linked with disordered eating behaviour among females (Ansell et al., 2012). If one were to consider disordered eating and maladaptive interpersonal behaviour as expressions of the same underlying pathology, the current research has identified a novel preliminary framework to understand patterns of maladaptive behaviour. More specifically, an inverse relationship was found between PID-5 intimacy avoidance and binge eating. When considering that PID-5 submissiveness was also indirectly associated with binge eating, it can be implied that a desire to connect with others to the extent that one may be willing to sacrifice their own needs may be an interpersonal style associated with binge eating behaviour, (Ansell et al., 2012; Brugnera et al., 2019). An indirect relationship between being cautious around others and purging was found in our study, consistent with past research indicating that a suspicious interpersonal style may be associated with purging behaviour (Gilmartin et al., 2023). Social insensitivity has been found to be related to increased exercise among females (Dalle Grave et al., 2008) and the current study provides novel understanding by indication that PID-5 manipulativeness may be associated with excessive exercise behaviour.

The current study provided a quantitative example of Vandereycken and Van Humbeeck (2008)’s qualitative work by indicating that high risk taking was related to Restrictive eating behaviour. Given that restrictive eating has been found to put an individual at risk of several physical health complications (Mitchell and Crow, 2006), the current finding may reflect a tendency to dismiss potential risk behaviour in pursuit of aesthetic goals. In addition, the results revealed PID-5 Dishonesty as a predictor of binge eating, purging and chewing and spitting. The current research therefore may reflect secrecy and concealment as identified by Vandereycken and Van Humbeeck (2008). However, it is unclear if the relationship between PID-5 Dishonesty and disordered eating reflects dishonesty surrounding eating behaviour specifically or a reflection on broader interpersonal difficulties (Ansell et al., 2012; Brugnera et al., 2019).

Body dissatisfaction was found to mediate relationships that have previously been identified in past research, more specifically between a lack of pleasure and restrictive eating (Boehm et al., 2018; Dolan et al., 2022a,b) and anxiety and compulsive exercise (Weinstein et al., 2015). Age was only found to significantly predict restrictive eating behaviour, in that younger individuals were more likely to engage in restrained eating (American Psychiatric Association [APA], 2013; Puccio et al., 2017).

### Limitations

In interpreting the results of the current study, there are some limitations that should be considered. As there is variability in ED presentations between ethnic backgrounds (Rodgers et al., 2018) and between White and Indigenous Australians (Burt et al., 2020), it is notable that the majority of participants who completed the survey for the current study identified as White. In addition, the data gathered from gender diverse participants was excluded from the statistical analyses conducted in this study. Taken together, it is important to show caution when generalising the current results to other populations. It is also important to note that the path models for males did not demonstrate a good fit of the data and were statistically underpowered. Although our study is novel in our focus on personality and eating behaviours among males, the results need to be interpreted with caution. The current data limited to being collected in Australia during the COVID-19 pandemic. The pandemic has been associated with increased psychological distress and eating disordered behaviours (Miller et al., 2021) and it is unclear if and when these factors will return to pre-pandemic levels. In addition, only a small to moderate proportion of the prospective participants for the study completed the survey. Whilst we cannot be sure why this is, we do know that most of the participants who dropped out did so early in the survey. It can be assumed that they anticipated fatigue at the length of the survey progress bar. Finally, the current study’s cross-sectional design does not allow us to hypothesise how personality and eating behaviours may predict one another over time. In addition, the cross-sectional design makes drawing conclusions from a mediation model ambiguous. However, this study may provide a solid foundation for a future longitudinal study for this purpose.

### Implications and areas for future research

Broadly, the results of our research add to the growing body of research that has suggested that personality traits are related to eating behaviours (Dufresne et al., 2020; Gilmartin et al., 2022). Although there is a possibility that there are psychological differences in disordered eating behaviours between clinical and non-clinical samples, there are some tentative clinical implications that can be drawn. For example, it can potentially be implied that understanding how an individual’s personality traits are expressed through disordered eating behaviour may assist in selecting appropriate prevention or early intervention strategies that target an individual’s maladaptive expression of personality traits and draw on potential strengths. For example, interventions to improve social skills may be appropriate for males who engage in muscle building behaviour, and strategies to increase concern about potential risks while drawing on goal-focussed strengths may be helpful for males who engage in compulsive exercise or body building behaviour. As another example, strategies to improve concentration may be useful for females who engage in binge eating, and directly targeting dishonesty may be a useful intervention among females who binge eat, purge or chew and spit. In general, the current study provides further evidence that personality and disordered eating have a different relationship among males compared to females (Gilmartin et al., 2023). Therefore, it is important to be cautious about generalising what we know about females with eating disorders to males and people of other genders.

The results of the present study have identified several areas for further exploration. First, a number of differences were identified between the results reported in this paper, and the results reported in our previous research (Gilmartin et al., 2023) which explored the relationship between disordered eating and the FFM using the same sample. For example, the statistical models reported in the current study did not identify any personality traits associated with some disordered behaviours, such as chewing and spitting in males and muscle building in females, whereas comparable models using the FFM did identify significant predictors (Gilmartin et al., 2023). The AMPD and associated PID-5 has been mostly found to represent maladaptive variants of FFM traits (Thomas et al., 2013; Watson et al., 2013; James et al., 2015; Fowler et al., 2017), with the exception of the Openness FFM domain and the PID-5 Psychoticism domain, where the research has been mixed (De Fruyt et al., 2013; Quilty et al., 2013; Watson et al., 2013; Suzuki et al., 2015; Fowler et al., 2017). The differences between this current paper and our other research (Gilmartin et al., 2023) may reflect the alternate functions of the constructs, in that the PID-5 represents maladaptive variants of normative FFM traits. Alternatively, there are differences in the facet level structure between the FFM and PID-5 (Griffin and Samuel, 2014), and this may be reflected in the differences between this paper and Gilmartin et al. (2023), although further research is required. Finally, while our research has highlighted the complexity of the relationship between personality and disordered eating in a non-clinical sample, we believe that further research would benefit from a more thorough understanding of how clusters of personality traits relate to eating pathology in a non-clinical sample (e.g., Wildes et al., 2011; Jennings et al., 2021).

In general, as there are very few studies exploring the relationships between the PID-5 and disordered eating and EDs, further research is required to understand the relationships in broader samples. However, some tentative clinical implications can be drawn. Consistent with past research that has identified a relationship between EDs and Borderline Personality Disorder, traits indicative of Borderline Personality Disorder were found to predict binge eating in males (Impulsivity) and restrictive eating in females (Risk taking; Zanarini et al., 2010; American Psychiatric Association [APA], 2013; Farstad et al., 2016). As previous research has identified Avoidant Personality Disorder and Obsessive-Compulsive Personality Disorder as the most frequently occurring personality disorders among individuals with EDs, it was surprising that personality traits that are representative of these diagnoses such as Intimacy Avoidance, Perseveration, Withdrawal and Rigid Perfectionism were not as predictive in the current statistical models. The first possible explanation is that a measure of pathological personality dimensions such as the PID-5 may not detect subclinical traits in non-clinical populations. As noted above, past research has suggested that personality traits in non-clinical populations can be more adequately assessed by the PID-5’s normative counterpart, the FFM (Suzuki et al., 2015). As a second explanation, research has indicated that the PID-5 may not serve as a sufficient strategy of detecting Obsessive-Compulsive Personality Disorder or Avoidant Personality Disorder (Watson et al., 2013; Rojas and Widiger, 2017). There has been found to be low convergent validity for the rigid perfectionism scale in the PID-5 with other scales sought to measure traits associated with Obsessive-Compulsive Personality Disorder (Watson et al., 2013). Therefore, the Intimacy Avoidance, Perseveration, Withdrawal and Rigid Perfectionism scales may not have detected pathological traits that were associated with disordered eating.

## Conclusion

The overall aim of our research was to explore the relationship between the PID-5 pathological trait dimensions and six disordered eating behaviours among adolescent and young adult males and females. The findings indicate a series of unique personality profiles that differed dependent on gender and disordered eating behaviour. The current research provides an important extension to existing literature that has indicated disordered eating behaviours may serve as unique expressions of pathological personality traits. Furthermore, the present study has provided a basis for the understanding of less-studied disordered eating behaviours such as chewing and spitting, excessive exercise and muscle building among a community-based sample of males and females. In general, the results of the current study support assertions of the need to understand disordered eating behaviour in the context of personality pathology to enhance the formulation of potentially risky behaviour, and open several avenues for future research.

## Data availability statement

The raw data supporting the conclusions of this article will be made available by the authors, without undue reservation.

## Ethics statement

The studies involving human participants were reviewed and approved by the Monash University Human Research Ethics Committee. The patients/participants provided their written informed consent to participate in this study.

## Author contributions

TG, CG, and GS contributed to the conception and design of the study. TG performed statistical analyses and wrote the first draft of the manuscript. JD assisted with designing a statistical plan of the study. All authors contributed to manuscript revision, read, and approved the submitted version.
