# Supplementary material for: Dimensional personality pathology and disordered eating in young adults: measuring the DSM-5 alternative model using the PID-5
Source: Front Psychol. 2023 Jun 26;14:1113142. doi: 10.3389/fpsyg.2023.1113142 (PMC10330766; doi:10.3389/fpsyg.2023.1113142)
Supplement: Supplementary file 1 [file Data_Sheet_1.docx]

**Dimensional Personality Pathology and Disordered Eating in Australian Young Adults: Measuring the DSM-5 Alternative model using the PID-5**

**Supplementary Documentation**

Table of Contents

[Preliminary Analyses 3](#_Toc120018724)

[Confirmatory Factor Analysis (Step 1) 7](#_Toc120018725)

[Restrictive Eating Behaviour 8](#_Toc120018726)

[Step 2: Path model results for each PID-5 domain 8](#_Toc120018727)

[Step 3: Results for composite path model 9](#_Toc120018728)

[Step 4: Composite path model predicting Body Dissatisfaction 10](#_Toc120018729)

[Binge Eating Behaviour 11](#_Toc120018730)

[Step 2: Path model results for each PID-5 domain 11](#_Toc120018731)

[Step 3: Results for composite path model 12](#_Toc120018732)

[Step 4: Composite path model predicting Body Dissatisfaction 13](#_Toc120018733)

[Purging Behaviour 14](#_Toc120018734)

[Step 2: Path model results for each PID-5 domain 14](#_Toc120018735)

[Step 3: Results for composite path model 15](#_Toc120018736)

[Step 4: Composite path model predicting Body Dissatisfaction 16](#_Toc120018737)

[Chewing and Spitting Behaviour 17](#_Toc120018738)

[Step 2: Path model results for each PID-5 domain 17](#_Toc120018739)

[Step 3: Results for composite path model 18](#_Toc120018740)

[Step 4: Composite path model predicting Body Dissatisfaction 18](#_Toc120018741)

[Excessive Exercise 20](#_Toc120018742)

[Step 2: Path model results for each PID-5 domain 20](#_Toc120018743)

[Step 3: Results for composite path model 21](#_Toc120018744)

[Step 4: Composite path model predicting Body Dissatisfaction 22](#_Toc120018745)

[Muscle Building 23](#_Toc120018746)

[Step 2: Path model results for each PID-5 domain 23](#_Toc120018747)

[Step 3: Results for composite path model 24](#_Toc120018748)

[Step 4: Composite path model predicting Body Dissatisfaction 24](#_Toc120018749)

# Preliminary Analyses

Table 1

Correlations between the EPSI subscales and the subscale scores on the PID-5, DASS and EDE-QS

|  | Body Dissatisfaction | | Binge Eating | | Purging | | Restricting | | Excessive Exercise | | Muscle Building | | Chew qnss | |
| --- | --- | --- | --- | --- | --- | --- | --- | --- | --- | --- | --- | --- | --- | --- |
|  | Males | Females | Males | Females | Males | Females | Males | Females | Males | Females | Males | Females | Males | Females |
| Body Dissatisfaction | 1 | 1 |  |  |  |  |  |  |  |  |  |  |  |  |
| Binge Eating | 0.53** | 0.34** | 1 | 1 |  |  |  |  |  |  |  |  |  |  |
| Purging | 0.45** | 0.54** | 0.31** | 0.33** | 1 | 1 |  |  |  |  |  |  |  |  |
| Restricting | 0.18* | 0.29** | -0.04 | -0.10* | 0.22** | 0.35** | 1 | 1 |  |  |  |  |  |  |
| Excessive Exercise | 0.10 | 0.31** | 0.14 | 0.11* | 0.16* | 0.33** | -0.10 | 0.23** | 1 | 1 |  |  |  |  |
| Muscle Building | 0.20** | 0.17** | 0.27** | 0.19** | 0.32** | 0.27** | 0.10 | 0.13* | 0.59** | 0.49** | 1 | 1 |  |  |
| Chew qnss | 0.19* | 0.33** | 0.18* | 0.18** | 0.64** | 0.58** | 0.22** | 0.33** | 0.12 | 0.30** | 0.27** | 0.12* | 1 | 1 |
| EDE QS total | 0.69** | 0.48** | 0.47** | 0.24** | 0.52** | 0.38** | 0.04 | 0.31** | 0.29** | 0.18** | 0.24** | 0.18** | 0.37** | 0.26** |
| Anhedonia | 0.35** | 0.42** | 0.27** | 0.20** | 0.09 | 0.31** | 0.17* | 0.35** | 0.01 | 0.24** | 0.06 | 0.11* | 0.0 | 0.18** |
| Anxiousness | 0.30** | 0.07 | 0.26** | 0.21** | 0.07 | 0.08 | 0.16* | 0.10 | -0.05 | 0.12* | -0.08 | 0.14** | 0.01 | 0.12* |
| Attention Seeking | 0.16* | 0.11* | 0.25** | 0.17** | 0.17* | 0.16** | 0.14 | 0.06 | 0.06 | 0.11* | 0.16* | 0.08 | 0.15 | 0.14** |
| Callousness | 0.05 | 0.23** | 0.12 | 0.37** | 0.17* | 0.32** | 0.21** | 0.20** | 0.14 | 0.19** | 0.28** | 0.17** | 0.09 | 0.28** |
| Deceitfulness | 0.16* | 0.45** | 0.16* | 0.18** | 0.17* | 0.37** | 0.17* | 0.35** | 0.04 | 0.17** | 0.09 | 0.21** | 0.13 | 0.20** |
| Depressivity | 0.38** | 0.28** | 0.32** | 0.26** | 0.19* | 0.23** | 0.19* | 0.19** | 0.01 | 0.05 | 0.08 | 0.15** | 0.09 | 0.09 |
| Distractability | 0.23** | 0.30** | 0.22** | 0.16** | 0.14 | 0.30** | 0.12 | 0.37** | -0.14 | 0.20** | -0.06 | 0.15** | 0.05 | 0.20** |
| Eccentricity | 0.15 | 0.36** | 0.10 | 0.27** | 0.08 | 0.29** | 0.24** | 0.32** | 0.015 | 0.13** | 0.08 | 0.14** | -0.02 | 0.15** |
| Emotional Lability | 0.39** | -0.08 | 0.40** | 0.21** | 0.26** | 0.02 | 0.19* | -0.01 | -0.01 | 0.07 | -0.03 | 0.10* | 0.09 | 0.07 |
| Grandiosity | 0.12 | 0.31** | 0.15 | 0.28** | 0.16* | 0.29** | 0.08 | 0.24** | 0.19* | 0.21** | 0.22** | 0.09 | 0.12 | 0.23** |
| Hostility | 0.30** | 0.29** | 0.32** | 0.28** | 0.15* | 0.33** | 0.11 | 0.28** | 0.05 | 0.13* | 0.05 | 0.17** | 0.07 | 0.26** |
| Impulsivity | 0.34** | 0.08 | 0.46** | -0.07 | 0.13 | 0.10* | 0.17* | 0.20** | 0.13 | 0.06 | 0.14 | 0.02 | 0.01 | 0.09 |
| Intimacy Avoidance | -0.01 | 0.21** | -0.06 | 0.29** | 0.11 | 0.25** | 0.00 | 0.18** | -0.05 | 0.13** | 0.03 | 0.15** | 0.00 | 0.18** |
| Irresponsibility | 0.30** | 0.16** | 0.35** | 0.25** | 0.30** | 0.23** | 0.16* | 0.16** | 0.03 | 0.20** | 0.11 | 0.14** | 0.25** | 0.16** |
| Manipulativeness | 0.10 | 0.24** | 0.14 | 0.23** | 0.11 | 0.32** | 0.10 | 0.31** | 0.19* | 0.24** | 0.19* | 0.16** | 0.02 | 0.17** |
| Perceptual Dysregulation | 0.31** | 0.32** | 0.20** | 0.25** | 0.36** | 0.25** | 0.17* | 0.29** | -0.02 | 0.20** | 0.04 | 0.17** | 0.25** | 0.23** |
| Perseveration | 0.36** | 0.09 | 0.37** | 0.05 | 0.18* | 0.10* | 0.16* | 0.05 | -0.01 | 0.05 | 0.04 | -0.05 | 0.11 | 0.13** |
| Restricted Affectivity | -0.03 | 0.33** | -0.10 | 0.09 | -0.04 | 0.26** | 0.15 | 0.35** | 0.12 | 0.30** | 0.20** | 0.09 | -0.09 | 0.18** |
| Rigid Perfectionism | 0.18* | 0.20** | 0.18* | 0.16** | 0.11 | 0.33** | 0.05 | 0.30** | -0.00 | 0.15** | -0.04 | 0.17** | 0.02 | 0.23** |
| Risk Taking | 0.24** | 0.31** | 0.23** | 0.16** | 0.20** | 0.24** | 0.20* | 0.28** | 0.28** | 0.14** | 0.25** | 0.13* | 0.09 | 0.15** |
| Separation Insecurity | 0.30** | 0.25** | 0.28** | 0.19** | 0.16* | 0.19** | 0.19* | 0.18** | 0.08 | 0.09 | 0.10 | 0.06 | 0.05 | 0.08 |
| Submissiveness | 0.25** | 0.32** | 0.28** | 0.24** | 0.02 | 0.37** | 0.08 | 0.34** | -0.14 | 0.16** | -0.09 | 0.17** | 0.03 | 0.11* |
| Suspiciousness | 0.36** | 0.20** | 0.27** | 0.22** | 0.24** | 0.32** | 0.21** | 0.29** | 0.06 | 0.17** | 0.12 | 0.19** | 0.06 | 0.09 |
| Unusual Beliefs and Experiences | 0.23** | 0.20** | 0.17* | 0.09 | 0.31** | 0.22** | 0.22** | 0.23** | 0.10 | 0.08 | 0.11 | 0.12* | 0.18* | 0.10 |
| Withdrawal | 0.23** | 0.45** | 0.08 | 0.26** | 0.07 | 0.35** | 0.13 | 0.39** | -0.02 | 0.21** | 0.07 | 0.16** | -0.02 | 0.20** |
| Negative Affect | 0.41** | 0.33** | 0.39** | 0.11* | 0.20** | 0.30** | 0.22** | 0.32** | 0.01 | 0.14** | -0.01 | 0.13** | 0.06 | 0.20** |
| Detachment | 0.25** | 0.21** | 0.13 | 0.35** | 0.12 | 0.25** | 0.13 | 0.18** | -0.03 | 0.15** | 0.07 | 0.19** | -0.00 | 0.15** |
| Antagonism | 0.22** | 0.33** | 0.25** | 0.34** | 0.20* | 0.34** | 0.15 | 0.27** | 0.11 | 0.12* | 0.16* | 0.19** | 0.09 | 0.22** |
| Disinhibition | 0.36** | 0.30** | 0.43** | 0.24** | 0.23** | 0.37** | 0.19* | 0.39** | 0.01 | 0.25** | 0.07 | 0.20** | 0.11 | 0.19** |
| Psychoticism | 0.27** | 0.47** | 0.18* | 0.17** | 0.28** | 0.35** | 0.27** | 0.32** | 0.04 | 0.14** | 0.09 | 0.17** | 0.15 | 0.23** |
| DASS Depression | 0.39** | 0.41** | 0.29** | 0.18** | 0.26** | 0.41** | 0.16* | 0.44** | 0.11 | 0.22** | 0.15* | 0.10* | 0.18* | 0.26** |
| DASS Anxiety | 0.39** | 0.45** | 0.38** | 0.20** | 0.30** | 0.36** | 0.08 | 0.35** | 0.10 | 0.24** | 0.14 | 0.15** | 0.20* | 0.24** |
| DASS Stress | 0.37** | 0.50** | 0.37** | 0.21** | 0.30** | 0.42** | 0.03 | 0.42** | 0.06 | 0.22** | 0.07 | 0.16** | 0.25** | 0.27** |
| DASS Total | 0.42** | 0.73** | 0.38** | 0.43** | 0.31** | 0.70** | 0.10 | 0.29** | 0.10 | 0.52* | 0.13 | 0.28** | 0.23** | 0.50** |

**p*<0.05, ***p*<0.001

Table 2

Means and Standard Deviations for age, EPSI, PID-5, DASS and EDE-QS

|  | |  | | | Males  N = 167 | | Females  N = 394 | | Gender Diverse  N = 10 | |
| --- | --- | --- | --- | --- | --- | --- | --- | --- | --- | --- |
|  | |  | | | M | SD | M | SD | M | SD |
| Age | | |  | | 21.76 | 3.618 | 22.307 | 3.94 | 21.80 | 3.048 |
| EPSI | | |  | |  |  |  |  |  |  |
|  | Body Dissatisfaction | | | | 10.58 | 7.00 | 16.54 | 7.09 | 12.10 | 7.22 |
|  | Restricting | | | | 7.30 | 5.86 | 9.07 | 6.29 | 8.60 | 6.52 |
|  | Binge Eating | | | | 11.44 | 7.02 | 13.08 | 7.90 | 12.70 | 10.37 |
|  | Purging | | | | 1.49 | 3.83 | 3.28 | 5.06 | 2.30 | 7.27 |
|  | Chew and spit | | | | 0.23 | 0.72 | 0.36 | 0.88 | 0.10 | 0.32 |
|  | Excessive Exercise | | | | 8.32 | 5.12 | 6.94 | 5.51 | 6.10 | 5.04 |
|  | Muscle Building | | | | 5.52 | 4.86 | 2.88 | 3.41 | 3.20 | 2.86 |
| PID-5 | |  | | |  |  |  |  |  |  |
|  | Anhedonia | | | | 1.07 | 0.84 | 1.13 | 0.85 | 1.03 | 0.81 |
|  | Anxiousness | | | | 1.59 | 0.87 | 1.99 | 0.81 | 1.98 | 0.84 |
|  | Attention Seeking | | | | 1.22 | 0.80 | 1.15 | 0.80 | 1.13 | 0.81 |
|  | Callousness | | | | 0.50 | 0.59 | 0.29 | 0.47 | 0.35 | 0.56 |
|  | Deceitfulness | | | | 0.79 | 0.62 | 0.65 | 0.63 | 0.93 | 0.78 |
|  | Depressivity | | | | 0.87 | 0.88 | 0.99 | 0.92 | 1.08 | 1.12 |
|  | Distractability | | | | 1.39 | 0.71 | 1.42 | 0.75 | 1.48 | 0.77 |
|  | Eccentricity | | | | 1.44 | 0.83 | 1.33 | 0.87 | 2.15 | 1.13 |
|  | Emotional Lability | | | | 0.85 | 0.85 | 1.40 | 0.86 | 1.08 | 1.09 |
|  | Grandiosity | | | | 0.60 | 0.63 | 0.39 | 0.52 | 0.23 | 0.42 |
|  | Hostility | | | | 0.91 | 0.77 | 1.10 | 0.78 | 0.80 | 0.67 |
|  | Impulsivity | | | | 0.97 | 0.69 | 0.95 | 0.76 | 0.98 | 0.68 |
|  | Intimacy Avoidance | | | | 0.93 | 0.79 | 0.85 | 0.85 | 0.98 | 1.16 |
|  | Irresponsibility | | | | 0.50 | 0.53 | 0.53 | 0.54 | 0.78 | 0.52 |
|  | Manipulativeness | | | | 0.95 | 0.67 | 0.73 | 0.64 | 1.13 | 1.01 |
|  | Perceptual Dysregulation | | | | 0.45 | 0.56 | 0.53 | 0.63 | 0.88 | 0.67 |
|  | Perseveration | | | | 1.21 | 0.73 | 1.37 | 0.71 | 1.30 | 0.66 |
|  | Restricted Affectivity | | | | 1.43 | 0.76 | 0.97 | 0.74 | 1.33 | 1.10 |
|  | Rigid Perfectionism | | | | 1.26 | 0.79 | 1.43 | 0.82 | 1.48 | 0.66 |
|  | Risk Taking | | | | 0.78 | 0.68 | 0.68 | 0.67 | 0.95 | 0.67 |
|  | Separation Insecurity | | | | 1.26 | 0.84 | 1.32 | 0.85 | 1.18 | 1.00 |
|  | Submissiveness | | | | 1.45 | 0.68 | 1.66 | 0.67 | 1.38 | 0.72 |
|  | Suspiciousness | | | | 0.82 | 0.67 | 0.92 | 0.73 | 1.08 | 0.67 |
|  | Unusual Beliefs and Experiences | | | | 0.60 | 0.59 | 0.64 | 0.69 | 1.25 | 0.94 |
|  | Withdrawal | | | | 1.02 | 0.73 | 1.09 | 0.69 | 1.38 | 0.96 |
|  | Negative Affect | | | | 1.23 | 0.69 | 1.57 | 0.68 | 1.41 | 0.69 |
|  | Detachment | | | | 1.00 | 0.61 | 1.02 | 0.61 | 1.13 | 0.83 |
|  | Antagonism | | | | 0.98 | 0.45 | 0.85 | 0.44 | 0.94 | 0.62 |
|  | Disinhibition | | | | 0.95 | 0.52 | 0.97 | 0.55 | 1.08 | 0.50 |
|  | Psychoticism | | | | 0.83 | 0.53 | 0.84 | 0.61 | 1.43 | 0.80 |
| DASS | |  | | |  |  |  |  |  |  |
|  | DASS Total | | | | 46.86 | 32.80 | 53.56 | 30.48 | 17.20 | 13.34 |
|  | Depression | | | | 16.74 | 12.91 | 17.88 | 12.74 | 18.20 | 12.91 |
|  | Anxiety | | | | 13.95 | 11.95 | 15.79 | 11.28 | 19.60 | 8.63 |
|  | Stress | | | | 16.17 | 11.10 | 19.89 | 10.59 | 55.00 | 30.71 |
| EDE-Q TOTAL | | | |  | 9.28 | 7.36 | 13.34 | 9.26 | 9.00 | 10.22 |

# Confirmatory Factor Analysis (Step 1)

Table 3

Model Fit Statistics for Confirmatory Factor Analysis of the PID-5

|  | χ^2^(*df*) | CFI | TLI | RMSEA (90% CI) |
| --- | --- | --- | --- | --- |
| PID-5 | 1875.16**(261) | 0.769 | 0.734 | 0.104 (0.100 - 0.109) |

**p*<0.05, ***p*<0.001

Table 4

*Factor Loadings for each of the PID-5 facets and comparison with (Maples et al., 2015)*

|  | |  | Coefficient | Comparison data |
| --- | --- | --- | --- | --- |
| Negative Affect | |  |  |  |
|  | Emotional lability | | 0.76 | 0.65 |
|  | Anxiousness | | 0.71 | 0.74 |
|  | Separation Insecurity | | 0.49 | 0.66 |
|  | Submissiveness | | 0.44 | 0.47 |
|  | Hostility | | 0.65 | 0.41 |
|  | Perseveration | | 0.71 | 0.56 |
| Detachment | |  |  |  |
|  | Withdrawal | | 0.55 | 0.78 |
|  | Intimacy avoidance | | 0.34 | 0.54 |
|  | Anhedonia | | 0.90 | 0.60 |
|  | Depressivity | | 0.92 | 0.56 |
|  | Restricted affect | | 0.22 | 0.54 |
|  | Suspiciousness | | 0.65 | 0.39 |
| Antagonism | |  |  |  |
|  | Manipulativeness | | 0.77 | 0.66 |
|  | Deceitfulness | | 0.86 | 0.60 |
|  | Grandiosity | | 0.55 | 0.70 |
|  | Attention seeking | | 0.47 | 0.56 |
|  | Callousness | | 0.54 | 0.53 |
| Disinhibition | |  |  |  |
|  | Irresponsibility | | 0.66 | 0.48 |
|  | Impulsivity | | 0.64 | 0.74 |
|  | Distractibility | | 0.59 | 0.52 |
|  | Risk taking | | 0.51 | 0.59 |
|  | Rigid perfectionism | | 0.38 | -.02 |
| Psychoticism | |  |  |  |
|  | Unusual beliefs and experiences | | 0.75 | 0.77 |
|  | Eccentricity | | 0.64 | 0.38 |
|  | Cognitive and perceptual dysregulation | | 0.79 | 0.67 |

Coefficient = Standardised regression weights.

Generally, factor loadings were acceptable. Where factor loadings were unfavourable, there appeared to be some consistency with the findings of other studies that have explored the structure of the PID-5.

# Restrictive Eating Behaviour

## Step 2: Path model results for each PID-5 domain

Table 5

*Path model fit statistics for each PID-5 domain as predictors of restrictive eating behaviour*

|  | Males | | | |  | Females | | | |
| --- | --- | --- | --- | --- | --- | --- | --- | --- | --- |
|  | χ^2^ (*df*) | CFI | TLI | RMSEA  (90% CI) |  | χ^2^(*df*) | CFI | TLI | RMSEA  (90% CI) |
| Negative Affect | 12.40 (7) | 0.984 | 0.936 | 0.068  (0.000 – 0.129) |  | 2.77 (3) | 1.000 | 1.003 | 0.000  (0.000 - 0.083) |
| Detachment | 13.48**(1) | 0.968 | 0.513 | 0.274  (0.157 – 0.413) |  | 8.62* (1) | 0.992 | 0.876 | 0.139  (0.065 – 0.231) |
| Antagonism | 0.750 (1) | 1.000 | 1.017 | 0.000  (0.000 – 0.195) |  | 17.58**(1) | 0.973 | 0.598 | 0.207  (0.130 – 0.296) |
| Disinhibition | 2.50 (2) | 0.996 | 0.974 | 0.039  (0.000 – 0.164) |  | 5.34 (3) | 0.996 | 0.978 | 0.045  (0.000 – 0.105) |
| Psychoticism | 79.62**(1) | 0.373 | -2.759 | 0.688  (0.565 – 0.820) |  | 108.75**(1) | 0.752 | -0.257 | 0.369  (0.311 – 0.429) |

**p*<0.05, ***p*<0.001

Table 6

*Standardised coefficients for each PID-5 domain as a predictor of restrictive eating behaviour within domain path analyses for males and females*

|  | |  | Males | |  | Females | |
| --- | --- | --- | --- | --- | --- | --- | --- |
|  | |  | Coefficient | *p* |  | Coefficient | *p* |
| Negative Affect | |  |  |  |  |  |  |
|  | Emotional lability | | 0.15 | 0.163 |  | **0.18** | **0.010** |
|  | Anxiousness | | 0.02 | 0.863 |  | **0.16** | **0.008** |
|  | Separation Insecurity | | 0.14 | 0.086 |  | 0.11 | 0.053 |
|  | Submissiveness | | 0.01 | 0.935 |  | 0.02 | 0.722 |
|  | Hostility | | -0.01 | 0.946 |  | -0.02 | 0.741 |
|  | Perseveration | | 0.00 | 0.977 |  | 0.08 | 0.170 |
|  | Restricted Affectivity | | **0.21** | **0.006** |  | 0.08 | 0.098 |
| Detachment | |  |  |  |  |  |  |
|  | Withdrawal | | 0.06 | 0.485 |  | -0.01 | 0.909 |
|  | Intimacy avoidance | | -0.10 | 0.238 |  | **0.11** | **0.030** |
|  | Anhedonia | | 0.01 | 0.936 |  | -0.00 | 0.982 |
|  | Depressivity | | 0.07 | 0.641 |  | **0.20** | **0.026** |
|  | Suspiciousness | | 0.16 | 0.110 |  | **-0.22** | **<0.001** |
| Antagonism | |  |  |  |  |  |  |
|  | Manipulativeness | | -0.03 | 0.783 |  | 0.09 | 0.204 |
|  | Deceitfulness | | 0.08 | 0.446 |  | **0.09** | **0.008** |
|  | Grandiosity | | -0.13 | 0.175 |  | **-0.15** | **0.014** |
|  | Attention seeking | | 0.16 | 0.067 |  | 0.05 | 0.359 |
|  | Callousness | | **0.24** | **0.011** |  | -0.01 | 0.914 |
| Disinhibition |  | |  |  |  |  |  |
|  | Irresponsibility | | 0.11 | 0.193 |  | -0.04 | 0.479 |
|  | Impulsivity | | 0.06 | 0.564 |  | **0.15** | **0.011** |
|  | Distractibility | | 0.01 | 0.888 |  | 0.04 | 0.440 |
|  | Risk taking | | 0.15 | 0.069 |  | **0.19** | **0.001** |
|  | Rigid perfectionism | | -0.00 | 0.956 |  | **0.32** | **<0.001** |
| Psychoticism |  | |  |  |  |  |  |
|  | Unusual beliefs and experiences | | 0.12 | 0.159 |  | 0.08 | 0.186 |
|  | Eccentricity | | **0.18** | **0.034** |  | **0.27** | **<0.001** |
|  | Cognitive and perceptual dysregulation | | 0.04 | -0.635 |  | 0.11 | 0.068 |

Coefficient = Standardised regression weights**. Boldface** indicates a significant coefficient at the <0.05 level

## Step 3: Results for composite path model

Table 7

*Path model fit statistics to predict restrictive eating behaviour in males and females*

|  | χ^2^(*df*) | CFI | TLI | RMSEA (90% CI) |
| --- | --- | --- | --- | --- |
| Males | 10.88 (5) | 0.952 | 0.799 | 0.084 (0.000 - 0.153) |
| Females | 13.94**(8) | 0.996 | 0.968 | 0.043 (0.081 - 0.100) |

**p*<0.05, ***p*<0.001

Table 8

*Standardised coefficients for each PID-5 scale in the composite path model predicting restrictive eating behaviour for males and females*

|  | Males | |  | Females | |
| --- | --- | --- | --- | --- | --- |
|  | Coefficient | *p* |  | Coefficient | *p* |
| Anxiousness | - | *-* |  | 0.10 | 0.081 |
| Attention seeking | 0.06 | 0.414 |  | - | *-* |
| Callousness | 0.11 | 0.173 |  | - | *-* |
| Deceitfulness | - | *-* |  | 0.02 | 0.774 |
| Depressivity | - | *-* |  | -0.01 | 0.130 |
| Eccentricity | 0.12 | 0.152 |  | 0.11 | 0.051 |
| Emotional lability | - | *-* |  | 0.09 | 0.130 |
| Grandiosity | - | *-* |  | **-0.14** | **0.004** |
| Impulsivity | - | *-* |  | 0.09 | 0.137 |
| Intimacy avoidance | - | *-* |  | **0.10** | **0.036** |
| Restricted Affectivity | 0.10 | 0.213 |  | - | *-* |
| Rigid perfectionism | - | *-* |  | **0.20** | **<0.001** |
| Risk taking | 0.07 | 0.429 |  | **0.13** | **0.024** |
| Separation Insecurity | 0.14 | 0.076 |  | - | *-* |
| Suspiciousness | - | *-* |  | 0.05 | 0.381 |

Coefficient = Standardised regression weights**. Boldface** indicates a significant coefficient at the <0.05 level

## Step 4: Composite path model predicting Body Dissatisfaction

Table 9

*Restrictive eating Path model fit statistics to predict Body Dissatisfaction in males and females*

|  | χ^2^ (*df*) | CFI | TLI | RMSEA |
| --- | --- | --- | --- | --- |
| Males | 16.06 (9) | 0.949 | 0.840 | 0.069 (0.000 - 0.122) |
| Females | 22.78(16) | 0.996 | 0.979 | 0.033 (0.000 - 0.061) |

**p*<0.05, ***p*<0.001

Table 10

*Standardised coefficients for each PID-5 scale from the restrictive eating model and age as predictors of Body Dissatisfaction for males and females*

|  | Males | | |  | | Females | |
| --- | --- | --- | --- | --- | --- | --- | --- |
|  | Coefficient | *p* | |  | | Coefficient | *p* |
| Anxiousness | - | *-* | |  | | **0.17** | **0.003** |
| Attention seeking | 0.01 | 0.896 | |  | | - | *-* |
| Callousness | -0.05 | 0.525 | |  | | - | *-* |
| Deceitfulness | - | *-* | |  | | **0.14** | **0.006** |
| Depressivity | - | *-* | |  | | **0.23** | **<0.001** |
| Eccentricity | 0.06 | 0.438 | |  | | 0.04 | 0.453 |
| Emotional lability | - | *-* | |  | | 0.03 | 0.619 |
| Grandiosity | - | *-* | |  | | **-0.22** | **<0.001** |
| Impulsivity | - | *-* | |  | | **0.17** | **0.002** |
| Intimacy avoidance | - | *-* | |  | | -0.07 | 0.155 |
| Restricted Affectivity | -0.05 | 0.552 | |  | | - | *-* |
| Rigid perfectionism | - | *-* | |  | | **0.15** | **0.003** |
| Risk taking | **0.22** | **0.007** | |  | | -0.06 | 0.269 |
| Separation Insecurity | **0.26** | **0.001** | |  | | - | *-* |
| Suspiciousness | - | *-* | |  | | -0.02 | 0.787 |
| Age | 0.06 | 0439 |  | | -0.01 | | 0.788 |

Coefficient = Standardised regression weights**. Boldface** indicates a significant coefficient at the <0.05 level

# Binge Eating Behaviour

## Step 2: Path model results for each PID-5 domain

Table 11

*Path model fit statistics for each PID-5 domain as predictors of binge-eating behaviour*

|  | Males | | | |  | Females | | | |
| --- | --- | --- | --- | --- | --- | --- | --- | --- | --- |
|  | χ^2^ (*df*) | CFI | TLI | RMSEA  (90% CI) |  | χ^2^(*df*) | CFI | TLI | RMSEA  (90% CI) |
| Negative Affect | 12.40 (7) | 0.985 | 0.940 | 0.068  (0.000 – 0.129) |  | 2.77 (3) | 1.00 | 1.00 | 0.000  (0.000 - 0.083) |
| Detachment | 13.48**(1) | 0.969 | 0.532 | 0.274  (0.157 – 0.413) |  | 8.62* (1) | 0.99 | 0.87 | 0.139  (0.065 – 0.231) |
| Antagonism | 0.750 (1) | 1.000 | 1.017 | 0.000  (0.000 – 0.195) |  | 17.58**(1) | 0.98 | 0.62 | 0.207  (0.130 – 0.296) |
| Disinhibition | 2.50 (2) | 0.997 | 0.980 | 0.039  (0.000 – 0.164) |  | 5.34 (3) | 1.00 | 0.98 | 0.045  (0.000 – 0.105) |
| Psychoticism | 79.62**(1) | 0.346 | -2.923 | 0.688  (0.565 – 0.820) |  | 103.19**(1) | 0.74 | -0.58 | 0.51  (0.429 – 0.595) |

**p*<0.05, ***p*<0.001

Table 12

*Standardised coefficients for each PID-5 domain as a predictor of binge-eating behaviour within domain path analyses for males and females*

|  | |  | Males | |  | Females | |
| --- | --- | --- | --- | --- | --- | --- | --- |
|  | |  | Coefficient | *p* |  | Coefficient | *p* |
| Negative Affect | |  |  |  |  |  |  |
|  | Emotional lability | | 0.17 | 0.108 |  | 0.12 | 0.088 |
|  | Anxiousness | | -0.09 | 0.342 |  | -0.02 | 0.705 |
|  | Separation Insecurity | | 0.12 | 0.130 |  | 0.01 | 0.921 |
|  | Submissiveness | | **0.18** | **0.023** |  | **0.09** | **0.074** |
|  | Hostility | | 0.15 | 0.086 |  | 0.15 | 0.016 |
|  | Perseveration | | 0.13 | 0.175 |  | 0.10 | 0.104 |
|  | Restricted Affectivity | | -0.02 | 0.823 |  | 0.06 | 0.257 |
| Detachment | |  |  |  |  |  |  |
|  | Withdrawal | | -0.06 | 0.489 |  | -0.03 | 0.568 |
|  | Intimacy avoidance | | **-0.17** | **0.028** |  | **-0.14** | **0.010** |
|  | Anhedonia | | -0.06 | 0.866 |  | **0.27** | **0.003** |
|  | Depressivity | | 0.28 | 0.057 |  | -0.08 | 0.354 |
|  | Suspiciousness | | 0.14 | 0.124 |  | **0.17** | **0.005** |
| Antagonism | |  |  |  |  |  |  |
|  | Manipulativeness | | 0.01 | 0.888 |  | -0.03 | 0.608 |
|  | Deceitfulness | | 0.03 | 0.740 |  | **0.35** | **<0.001** |
|  | Grandiosity | | -0.00 | 0.994 |  | 0.06 | 0.308 |
|  | Attention seeking | | **0.23** | **0.008** |  | 0.08 | 0.126 |
|  | Callousness | | 0.08 | 0.389 |  | -0.05 | 0.426 |
| Disinhibition |  | |  |  |  |  |  |
|  | Irresponsibility | | **0.22** | **0.005** |  | **0.17** | **0.007** |
|  | Impulsivity | | **0.43** | **<0.001** |  | **0.17** | **0.007** |
|  | Distractibility | | -0.12 | 0.137 |  | **0.12** | **0.026** |
|  | Risk taking | | 0.02 | 0.800 |  | -0.06 | 0.291 |
|  | Rigid perfectionism | | 0.13 | 0.056 |  | 0.05 | 0.291 |
| Psychoticism |  | |  |  |  |  |  |
|  | Unusual beliefs and experiences | | 0.07 | 0.428 |  | 0.10 | 0.096 |
|  | Eccentricity | | 0.01 | 0.912 |  | 0.03 | 0.507 |
|  | Cognitive and perceptual dysregulation | | **0.16** | **0.037** |  | **0.14** | **0.037** |

Coefficient = Standardised regression weights**. Boldface** indicates a significant coefficient at the <0.05 level

## Step 3: Results for composite path model

Table 13

*Path model fit statistics to predict binge-eating behaviour in males and females*

|  | χ^2^(*df*) | CFI | TLI | RMSEA (90% CI) |
| --- | --- | --- | --- | --- |
| Males | 11.68*(5) | 0.979 | 0.880 | 0.090 (0.018 – 0.158) |
| Females | 1.18 (3) | 1.000 | 1.025 | 0.000 (0.000 – 0.058) |

**p*<0.05, ***p*<0.001

Table 14

*Standardised coefficients for each PID-5 scale in the composite path model predicting binge-eating behaviour for males and females*

|  | Males | |  | Females | |
| --- | --- | --- | --- | --- | --- |
|  | Coefficient | *p* |  | Coefficient | *p* |
| Anhedonia | - | *-* |  | 0.07 | 0.264 |
| Attention Seeking | 0.08 | 0.297 |  | - | *-* |
| Deceitfulness | - | *-* |  | **0.26** | **<0.001** |
| Depressivity | 0.08 | 0.379 |  | - | *-* |
| Distractibility | - | *-* |  | **0.20** | **0.001** |
| Hostility | 0.04 | 0.664 |  | 0.08 | 0.195 |
| Impulsivity | **0.31** | **<0.001** |  | 0.02 | 0.725 |
| Intimacy Avoidance | - | *-* |  | **-0.21** | **<0.001** |
| Irresponsibility | 0.13 | 0.123 |  | -0.00 | 0.968 |
| Perceptual Dysregulation | -0.05 | 0.554 |  | 0.04 | 0.452 |
| Rigid Perfectionism | 0.09 | 0.217 |  | - | *-* |
| Submissiveness | 0.13 | 0.073 |  | 0.09 | 0.071 |
| Suspiciousness | - | *-* |  | -0.03 | 0.620 |

Coefficient = Standardised regression weights**. Boldface** indicates a significant coefficient at the <0.05 level

## Step 4: Composite path model predicting Body Dissatisfaction

Table 15

*Binge-eating Path model fit statistics to predict Body Dissatisfaction in males and females*

|  | χ^2^ (*df*) | CFI | TLI | RMSEA |
| --- | --- | --- | --- | --- |
| Males | 31.59*(16) | 0.953 | 0.867 | 0.077 (0.036 – 0.116) |
| Females | 9.04 (11) | 1.000 | 1.008 | 0.000 (0.000 – 0.045) |

**p*<0.05, ***p*<0.001

Table 16

*Standardised coefficients for each PID-5 domain from the binge-eating behaviour model and age as predictors of Body Dissatisfaction for males and females*

|  | Males | |  | Females | |
| --- | --- | --- | --- | --- | --- |
|  | Coefficient | *p* |  | Coefficient | *p* |
| Anhedonia | - | *-* |  | **0.42** | **<0.001** |
| Attention Seeking | 0.03 | 0.698 |  | - | *-* |
| Deceitfulness | - | *-* |  | **0.11** | **0.047** |
| Depressivity | **0.19** | **0.032** |  | - | *-* |
| Distractibility | - | *-* |  | 0.07 | 0.256 |
| Hostility | 0.09 | 0.317 |  | 0.00 | 0.953 |
| Impulsivity | 0.15 | 0.084 |  | **0.16** | **0.005** |
| Intimacy Avoidance | - | *-* |  | -0.08 | 0.110 |
| Irresponsibility | -0.01 | 0.960 |  | **-0.14** | **0.026** |
| Perceptual Dysregulation | 0.13 | 0.141 |  | -0.01 | 0.823 |
| Rigid Perfectionism | 0.06 | 0.397 |  | - | *-* |
| Submissiveness | 0.10 | 0.166 |  | **0.10** | **0.036** |
| Suspiciousness | - | *-* |  | 0.01 | 0.904 |
| Age | -0.04 | 0.591 |  | -0.01 | 0.757 |

Coefficient = Standardised regression weights**. Boldface** indicates a significant coefficient at the <0.05 level

# Purging Behaviour

## Step 2: Path model results for each PID-5 domain

Table 17

*Path model fit statistics for each PID-5 domain as predictors of purging behaviour*

|  | Males | | | |  | Females | | | |
| --- | --- | --- | --- | --- | --- | --- | --- | --- | --- |
|  | χ^2^ (*df*) | CFI | TLI | RMSEA  (90% CI) |  | χ^2^(*df*) | CFI | TLI | RMSEA  (90% CI) |
| Negative Affect | 12.40 (7) | 0.984 | 0.935 | 0.068  (0.000 – 0.129) |  | 2.77 (3) | 1.000 | 1.003 | 0.000  (0.000 – 0.083) |
| Detachment | 13.48**(1) | 0.968 | 0.519 | 0.274  (0.157 – 0.413) |  | 8.62* (1) | 0.992 | 0.877 | 0.139  (0.065 – 0.231) |
| Antagonism | 0.750 (1) | 1.000 | 1.017 | 0.000  (0.000 – 0.195) |  | 17.85**(1) | 0.974 | 0.616 | 0.207  (0.130 – 0.296) |
| Disinhibition | 2.50 (2) | 0.997 | 0.976 | 0.039  (0.000 – 0.164) |  | 5.34 (3) | 0.996 | 0.978 | 0.045  (0.000 – 0.105) |
| Psychoticism | 79.62**(1) | 0.436 | -2.385 | 0.688  (0.565 – 0.820) |  | 103.19**(1) | 0.758 | -0.474 | 0.510  (0.429 – 0.595) |

**p*<0.05, ***p*<0.001

Table 18

*Standardised coefficients for each PID-5 domain as a predictor of purging behaviour within domain path analyses for males and females*

|  |  | | | | Males | |  | Females | |
| --- | --- | --- | --- | --- | --- | --- | --- | --- | --- |
|  |  | | | | Coefficient | *p* |  | Coefficient | *p* |
| Negative Affect | | | |  |  |  |  |  |  |
|  | Emotional lability | | | | **0.26** | **0.022** |  | 0.14 | 0.053 |
|  | Anxiousness | | | | -0.14 | 0.153 |  | 0.11 | 0.081 |
|  | Separation Insecurity | | | | 0.09 | 0.283 |  | 0.08 | 0.142 |
|  | Submissiveness | | | | -0.04 | 0.659 |  | 0.06 | 0.220 |
|  | Hostility | | | | -0.01 | 0.952 |  | 0.10 | 0.108 |
|  | Perseveration | | | | 0.08 | 0.444 |  | 0.03 | 0.645 |
|  | Restricted Affectivity | | | | 0.03 | 0.673 |  | 0.12 | **0.019** |
| Detachment | | |  | |  |  |  |  |  |
|  | Withdrawal | | | | -0.04 | 0.628 |  | -0.02 | 0.712 |
|  | Intimacy avoidance | | | | 0.06 | 0.448 |  | -0.01 | 0.820 |
|  | Anhedonia | | | | -0.25 | 0.074 |  | 0.17 | 0.054 |
|  | Depressivity | | | | 0.26 | 0.081 |  | 0.12 | 0.162 |
|  | Suspiciousness | | | | **0.20** | **0.038** |  | **0.22** | **<0.001** |
| Antagonism | |  | | |  |  |  |  |  |
|  | Manipulativeness | | | | -0.04 | 0.713 |  | 0.07 | 0.282 |
|  | Deceitfulness | | | | 0.08 | 0.447 |  | **0.32** | **<0.001** |
|  | Grandiosity | | | | 0.03 | 0.762 |  | **-0.18** | **0.002** |
|  | Attention seeking | | | | 0.13 | 0.124 |  | -0.01 | 0.802 |
|  | Callousness | | | | 0.12 | 0.219 |  | 0.06 | 0.284 |
| Disinhibition | | |  | |  |  |  |  |  |
|  | Irresponsibility | | | | **0.29** | **<0.001** |  | 0.03 | 0.581 |
|  | Impulsivity | | | | -0.05 | 0.610 |  | **0.19** | **0.002** |
|  | Distractibility | | | | -0.01 | 0.877 |  | 0.06 | 0.307 |
|  | Risk taking | | | | **0.18** | **0.031** |  | **0.16** | **0.005** |
|  | Rigid perfectionism | | | | 0.03 | 0.721 |  | **0.22** | **<0.001** |
| Psychoticism | |  | | |  |  |  |  |  |
|  | Unusual beliefs and experiences | | | | **0.19** | **0.017** |  | **0.15** | **0.012** |
|  | Eccentricity | | | | -0.10 | 0.205 |  | **0.16** | **0.002** |
|  | Cognitive and perceptual dysregulation | | | | **0.28** | **<0.001** |  | **0.13** | **0.038** |

Coefficient = Standardised regression weights**. Boldface** indicates a significant coefficient at the <0.05 level

## Step 3: Results for composite path model

Table 19

*Path model fit statistics to predict purging behaviour in males and females*

|  | χ^2^(*df*) | CFI | TLI | RMSEA (90% CI) |
| --- | --- | --- | --- | --- |
| Males | 6.45 (3) | 0.991 | 0.914 | 0.083 (0.000 – 0.173) |
| Females | 15.42 (13) | 0.995 | 0.995 | 0.043 (0.000 – 0.078) |

**p*<0.05, ***p*<0.001

Table 20

*Standardised coefficients for each PID-5 scale in the composite path model predicting purging behaviour for males and females*

|  | Males | |  | Females | |
| --- | --- | --- | --- | --- | --- |
|  | Coefficient | *p* |  | Coefficient | *p* |
| Anhedonia | -0.17 | 0.052 |  | - | *-* |
| Deceitfulness | - | *-* |  | **0.19** | **0.001** |
| Eccentricity | - | *-* |  | -0.00 | 0.993 |
| Emotional Lability | 0.14 | 0.111 |  | - | *-* |
| Grandiosity | - | *-* |  | **-0.17** | **<0.001** |
| Impulsivity | - | *-* |  | **0.15** | **0.013** |
| Irresponsibility | 0.11 | 0.257 |  | - | *-* |
| Perceptual Dysregulation | **0.22** | **0.028** |  | 0.03 | 0.650 |
| Restricted Affectivity | - | *-* |  | 0.03 | 0.489 |
| Rigid Perfectionism | - | *-* |  | **0.16** | **0.001** |
| Risk taking | **0.14** | **0.047** |  | 0.06 | 0.350 |
| Suspiciousness | 0.04 | 0.662 |  | **0.14** | **0.017** |
| Unusual Beliefs and Experiences | 0.08 | 0.398 |  | 0.09 | 0.175 |

Coefficient = Standardised regression weights**. Boldface** indicates a significant coefficient at the <0.05 level

## Step 4: Composite path model predicting Body Dissatisfaction

Table 21

*Purging Path model fit statistics to predict Body Dissatisfaction in males and females*

|  | χ^2^ (*df*) | CFI | TLI | RMSEA |
| --- | --- | --- | --- | --- |
| Males | 11.96 (7) | 0.988 | 0.936 | 0.065 (0.000 – 0.127) |
| Females | 27.09 (17) | 0.992 | 0.970 | 0.039 (0.000 – 0.065) |

**p*<0.05, ***p*<0.001

Table 22

*Standardised coefficients for each PID-5 scale from the purging behaviour model and age as predictors of Body Dissatisfaction for males and females*

|  | Males | |  | Females | |
| --- | --- | --- | --- | --- | --- |
|  | Coefficient | *p* |  | Coefficient | *p* |
| Anhedonia | 0.14 | 0.117 |  | - | *-* |
| Deceitfulness | - | *-* |  | **0.17** | **0.004** |
| Eccentricity | - | *-* |  | 0.09 | 0.109 |
| Emotional Lability | **0.22** | **0.009** |  | - | *-* |
| Grandiosity | - | *-* |  | **-0.27** | **<0.001** |
| Impulsivity | - | *-* |  | **0.21** | **<0.001** |
| Irresponsibility | 0.04 | 0.647 |  | - | *-* |
| Perceptual Dysregulation | 0.11 | 0.264 |  | -0.00 | 0.955 |
| Restricted Affectivity | - | *-* |  | 0.03 | 0.562 |
| Rigid Perfectionism | - | *-* |  | **0.26** | **<0.001** |
| Risk taking | **0.15** | **0.030** |  | -0.08 | 0.157 |
| Suspiciousness | 0.09 | 0.350 |  | **0.15** | **0.009** |
| Unusual Beliefs and Experiences | -0.04 | 0.659 |  | -0.02 | 0.739 |
| Age | -0.04 | 0.605 |  | 0.01 | 0.839 |

Coefficient = Standardised regression weights**. Boldface** indicates a significant coefficient at the <0.05 level

# Chewing and Spitting Behaviour

## Step 2: Path model results for each PID-5 domain

Table 23

*Path model fit statistics for each PID-5 scales as predictors of chewing and spitting behaviour*

|  | Males | | | |  | Females | | | |
| --- | --- | --- | --- | --- | --- | --- | --- | --- | --- |
|  | χ^2^ (*df*) | CFI | TLI | RMSEA  (90% CI) |  | χ^2^(*df*) | CFI | TLI | RMSEA  (90% CI) |
| Negative Affect | 12.38 (7) | 0.983 | 0.933 | 0.068  (0.000 – 0.129) |  | 2.77 (3) | 1.000 | 1.003 | 0.000  (0.000 – 0.083) |
| Detachment | 13.48**(1) | 0.967 | 0.506 | 0.274  (0.157 – 0.413) |  | 8.615*(1) | 0.991 | 0.870 | 0.139  (0.065 – 0.213) |
| Antagonism | 0.75 (1) | 1.000 | 1.017 | 0.000  (0.000 – 0.195) |  | 17.85**(1) | 0.974 | 0.605 | 0.207  (0.130 – 0.296) |
| Disinhibition | 2.50 (2) | 0.997 | 0.974 | 0.039  (0.004 – 0.164) |  | 5.34 (3) | 0.995 | 0.976 | 0.045  (0.000 – 0.105) |
| Psychoticism | 79.62**(1) | 0.346 | -2.923 | 0.688  (0.565 – 0.820) |  | 103.19**(1) | 0.734 | -0.594 | 0.510  (0.429 – 0.595) |

**p*<0.05, ***p*<0.001

Table 24

*Standardised coefficients for each PID-5 domain as a predictor of chewing and spitting behaviour within domain path analyses for males and females*

|  | |  | | | | Males | |  | Females | |
| --- | --- | --- | --- | --- | --- | --- | --- | --- | --- | --- |
|  | |  | | | | Coefficient | *p* |  | Coefficient | *p* |
| Negative Affect | | | |  | |  |  |  |  |  |
|  | Emotional lability | | | | | 0.02 | 0.866 |  | 0.00 | 0.999 |
|  | Anxiousness | | | | | -0.09 | 0.409 |  | 0.02 | 0.760 |
|  | Separation Insecurity | | | | | 0.02 | 0.857 |  | 0.05 | 0.348 |
|  | Submissiveness | | | | | 0.00 | 0.973 |  | -0.02 | 0.771 |
|  | Hostility | | | | | 0.03 | 0.777 |  | **0.14** | **0.034** |
|  | Perseveration | | | | | -0.11 | 0.286 |  | **0.13** | **0.027** |
|  | Restricted Affectivity | | | | | -0.08 | 0.339 |  | **0.11** | **0.033** |
| Detachment | | | |  | |  |  |  |  |  |
|  | Withdrawal | | | | | -0.07 | 0.457 |  | -0.04 | 0.468 |
|  | Intimacy avoidance | | | | | 0.00 | 0.987 |  | 0.03 | 0.530 |
|  | Anhedonia | | | | | -0.20 | 0.160 |  | **0.32** | **<0.001** |
|  | Depressivity | | | | | 0.28 | 0.076 |  | -0.05 | 0.603 |
|  | Suspiciousness | | | | | 0.02 | 0.876 |  | -0.02 | 0.704 |
| Antagonism | | | | |  |  |  |  |  |  |
|  | Manipulativeness | | | | | -0.12 | 0.211 |  | -0.04 | 0.595 |
|  | Deceitfulness | | | | | 0.11 | 0.266 |  | **0.30** | **<0.001** |
|  | Grandiosity | | | | | 0.04 | 0.717 |  | -0.10 | 0.106 |
|  | Attention seeking | | | | | 0.14 | 0.110 |  | 0.05 | 0.352 |
|  | Callousness | | | | | 0.04 | 0.674 |  | 0.04 | 0.482 |
| Disinhibition | | | |  | |  |  |  |  |  |
|  | Irresponsibility | | | | | **0.30** | **<0.001** |  | 0.04 | 0.474 |
|  | Impulsivity | | | | | -0.14 | 0.147 |  | **0.20** | **0.002** |
|  | Distractibility | | | | | -0.03 | 0.790 |  | -0.07 | 0.200 |
|  | Risk taking | | | | | 0.11 | 0.177 |  | 0.10 | 0.109 |
|  | Rigid perfectionism | | | | | -0.05 | 0.541 |  | **0.17** | **<0.001** |
| Psychoticism | | |  | | |  |  |  |  |  |
|  | Unusual beliefs and experiences | | | | | 0.07 | 0.428 |  | 0.09 | 0.137 |
|  | Eccentricity | | | | | 0.01 | 0.912 |  | **0.17** | **0.001** |
|  | Cognitive and perceptual dysregulation | | | | | **0.16** | **0.037** |  | **0.14** | **0.027** |

Coefficient = Standardised regression weights**. Boldface** indicates a significant coefficient at the <0.05 level

## Step 3: Results for composite path model

Table 25

*Path model fit statistics to predict chewing and spitting behaviour in males and females*

|  | χ^2^(*df*) | CFI | TLI | RMSEA (90% CI) |
| --- | --- | --- | --- | --- |
| Males | 29.91**(1) | 0.912 | 0.122 | 0.417 (0.297 – 0.552) |
| Females | 9.291 (6) | 0.997 | 0.975 | 0.037 (0.000 – 0.081) |

**p*<0.05, ***p*<0.001

Table 26

*Standardised coefficients for each PID-5 scale in the composite path model predicting chewing and spitting behaviour for males and females*

|  | Males | | | |  | | Females | | | |  |
| --- | --- | --- | --- | --- | --- | --- | --- | --- | --- | --- | --- |
|  | Coefficient | | *p* | |  | | Coefficient | | *p* | |  |
| Anhedonia | -0.22 | | 0.092 | |  | | 0.11 | | 0.078 | |  |
| Deceitfulness | | - | | *-* | |  | | **0.16** | | **0.003** | |
| Depressivity | | 0.11 | | 0.425 | |  | | - | | *-* | |
| Eccentricity | | - | | *-* | |  | | -0.03 | | 0.622 | |
| Hostility | | - | | *-* | |  | | 0.02 | | 0.748 | |
| Impulsivity | | - | | *-* | |  | | **0.15** | | **0.007** | |
| Irresponsibility | | **0.18** | | **0.041** | |  | | - | | *-* | |
| Perceptual Dysregulation | | **0.17** | | **0.046** | |  | | -0.02 | | 0.678 | |
| Perseveration | | - | | *-* | |  | | 0.05 | | 0.419 | |
| Restricted Affectivity | | - | | *-* | |  | | 0.08 | | 0.100 | |
| Rigid Perfectionism | | - | | *-* | |  | | 0.07 | | 0.183 | |

Coefficient = Standardised regression weights**. Boldface** indicates a significant coefficient at the <0.05 level

## Step 4: Composite path model predicting Body Dissatisfaction

Table 27

*Chewing and spitting path model fit statistics to predict Body Dissatisfaction in males and females*

|  | χ^2^ (*df*) | CFI | TLI | RMSEA |
| --- | --- | --- | --- | --- |
| Males | 4.19 (2) | 0.994 | 0.954 | 0.081 (0.000 – 0.092) |
| Females | 18.30 (13) | 0.995 | 0.979 | 0.032 (0.000 – 0.064) |

**p*<0.05, ***p*<0.001

Table 28

*Standardised coefficients for each PID-5 domain from the chewing and spitting behaviour model and age*

|  | | Females | |  | Males | | | | |
| --- | --- | --- | --- | --- | --- | --- | --- | --- | --- |
|  | | Coefficient | *p* |  | Coefficient | | | *p* | |
| Anhedonia | 0.11 | | 0.392 |  | **0.37** | | **<0.001** | |  |
| Deceitfulness | | - | *-* |  | | 0.04 | | 0.413 | |
| Depressivity | | 0.19 | 0.161 |  | | - | | *-* | |
| Eccentricity | | - | *-* |  | | -0.02 | | 0.375 | |
| Hostility | | - | *-* |  | | 0.05 | | 0.733 | |
| Impulsivity | | - | *-* |  | | **0.18** | | **<0.001** | |
| Irresponsibility | | 0.10 | 0.290 |  | | - | | *-* | |
| Perceptual Dysregulation | | 0.12 | 0.172 |  | | -0.07 | | 0.224 | |
| Perseveration | | - | *-* |  | | -0.01 | | 0.917 | |
| Restricted Affectivity | | - | *-* |  | | -0.04 | | 0.424 | |
| Rigid Perfectionism | | - | *-* |  | | **0.17** | | **<0.001** | |
| Age | | -0.06 | 0.403 |  | -0.02 | | | 0.619 | |

Coefficient = Standardised regression weights**. Boldface** indicates a significant coefficient at the <0.05 level

# Excessive Exercise

## Step 2: Path model results for each PID-5 domain

Table 29

*Path model fit statistics for each PID-5 domain as predictors of excessive exercise behaviour*

|  | Males | | | |  | Females | | | |
| --- | --- | --- | --- | --- | --- | --- | --- | --- | --- |
|  | χ^2^ (*df*) | CFI | TLI | RMSEA  (90% CI) |  | χ^2^(*df*) | CFI | TLI | RMSEA  (90% CI) |
| Negative Affect | 12.40 (7) | 0.984 | 0.934 | 0.068  (0.00 – 0.129) |  | 2.77 (3) | 1.000 | 1.003 | 0.000  (0.000 – 0.083) |
| Detachment | 13.48**(1) | 0.967 | 0.502 | 0.274  (0.157 – 0.413) |  | 8.62* (1) | 0.991 | 0.868 | 0.139  (0.065 – 0.231) |
| Antagonism | 0.75 (1) | 1.000 | 1.017 | 0.000  (0.000 – 0.195) |  | 17.85**(1) | 0.973 | 0.596 | 0.207  (0.130 – 0.296) |
| Disinhibition | 2.496 (2) | 0.997 | 0.976 | 0.039  (0.000 – 0.164) |  | 5.34 (3) | 0.995 | 0.976 | 0.045  (0.000 – 0.105) |
| Psychoticism | 79.620**(6) | 0.323 | -3.063 | 0.688  (0.565 – 0.820) |  | 103.19**(1) | 0.739 | -0.569 | 0.520  (0.429 – 0.595) |

**p*<0.05, ***p*<0.001

Table 30

*Standardised coefficients for each PID-5 domain as a predictor of excessive exercise behaviour within domain path analyses for males and females*

|  |  | | | | Males | |  | Females | |
| --- | --- | --- | --- | --- | --- | --- | --- | --- | --- |
|  |  | | | | Coefficient | *p* |  | Coefficient | *p* |
| Negative Affect | | |  | |  |  |  |  |  |
|  | Emotional lability | | | | -0.02 | 0.895 |  | -0.09 | 0.214 |
|  | Anxiousness | | | | -0.05 | 0.598 |  | **0.16** | **0.011** |
|  | Separation Insecurity | | | | 0.14 | 0.105 |  | 0.03 | 0.600 |
|  | Submissiveness | | | | -0.15 | 0.091 |  | -0.00 | 0.946 |
|  | Hostility | | | | 0.07 | 0.487 |  | **0.13** | **0.047** |
|  | Perseveration | | | | 0.02 | 0.881 |  | 0.09 | 0.123 |
|  | Restricted Affectivity | | | | 0.13 | 0.104 |  | 0.01 | 0.846 |
| Detachment | | |  | |  |  |  |  |  |
|  | Withdrawal | | | | -0.02 | 0.813 |  | -0.05 | 0.460 |
|  | Intimacy avoidance | | | | -0.06 | 0.472 |  | 0.02 | 0.678 |
|  | Anhedonia | | | | 0.03 | 0.841 |  | 0.13 | 0.156 |
|  | Depressivity | | | | -0.06 | 0.703 |  | 0.01 | 0.895 |
|  | Suspiciousness | | | | 0.11 | 0.283 |  | 0.10 | 0.121 |
| Antagonism | | | |  |  |  |  |  |  |
|  | Manipulativeness | | | | **0.22** | **0.019** |  | **0.14** | **0.046** |
|  | Deceitfulness | | | | -0.19 | 0.061 |  | 0.09 | 0.182 |
|  | Grandiosity | | | | 0.15 | 0.115 |  | -0.07 | 0.284 |
|  | Attention seeking | | | | -0.02 | 0.841 |  | 0.05 | 0.383 |
|  | Callousness | | | | 0.08 | 0.398 |  | 0.03 | 0.654 |
| Disinhibition | | |  | |  |  |  |  |  |
|  | Irresponsibility | | | | 0.06 | 0.460 |  | 0.09 | 0.145 |
|  | Impulsivity | | | | 0.14 | 0.141 |  | 0.05 | 0.431 |
|  | Distractibility | | | | **-0.28** | **0.002** |  | -0.09 | 0.103 |
|  | Risk taking | | | | **0.25** | **0.002** |  | 0.07 | 0.222 |
|  | Rigid perfectionism | | | | 0.02 | 0.827 |  | **0.29** | **<0.001** |
| Psychoticism | |  | | |  |  |  |  |  |
|  | Unusual beliefs and experiences | | | | **0.18** | **0.026** |  | -0.01 | 0.902 |
|  | Eccentricity | | | | -0.02 | 0.797 |  | **0.11** | **0.043** |
|  | Cognitive and perceptual dysregulation | | | | -0.13 | 0.097 |  | **0.18** | **0.005** |

Coefficient = Standardised regression weights**. Boldface** indicates a significant coefficient at the <0.05 level

## Step 3: Results for composite path model

Table 31

*Path model fit statistics to predict excessive exercise behaviour in males and females*

|  | χ^2^(*df*) | CFI | TLI | RMSEA (90% CI) |
| --- | --- | --- | --- | --- |
| Males | 4.27 (4) | 0.999 | 0.993 | 0.020 (0.000 – 0.121) |
| Females | 28.90*(1) | 0.990 | 0.722 | 0.142 (0.067 – 0.233) |

**p*<0.05, ***p*<0.001

Table 32

*Standardised coefficients for each PID-5 facet in the composite path model predicting excessive exercise behaviour for males and females*

|  | Males | |  | Females | |
| --- | --- | --- | --- | --- | --- |
|  | Coefficient | *p* |  | Coefficient | *p* |
| Anhedonia | - | *-* |  | -0.04 | 0.536 |
| Anxiousness | - | *-* |  | 0.09 | 0.169 |
| Deceitfulness | -0.11 | 0.284 |  | - | *-* |
| Distractibility | -0.19 | 0.033 |  | - | *-* |
| Eccentricity | - | *-* |  | -0.01 | 0.817 |
| Hostility | - | *-* |  | 0.04 | 0.542 |
| Manipulativeness | 0.16 | 0.085 |  | **0.11** | **0.027** |
| Perceptual Dysregulation | **-0.03** | **0.730** |  | **0.15** | **0.010** |
| Rigid Perfectionism | - | *-* |  | **0.20** | **<0.001** |
| Risk taking | **0.27** | **<0.001** |  | - | *-* |
| Submissiveness | **-0.02** | **0.858** |  | - | *-* |
| Unusual Beliefs and Experiences | 0.12 | 0.211 |  | - | *-* |

Coefficient = Standardised regression weights**. Boldface** indicates a significant coefficient at the <0.05 level

## Step 4: Composite path model predicting Body Dissatisfaction

Table 33

*Excessive exercise path model fit statistics to predict Body Dissatisfaction in males and females*

|  | χ^2^ (*df*) | CFI | TLI | RMSEA |
| --- | --- | --- | --- | --- |
| Females | 25.834 (19) | 0.990 | 0.991 | 0.030 (0.000 – 0.057) |
| Males | 9.945 (10) | 1.000 | 1.001 | 0.000 (0.000 – 0.084) |

**p*<0.05, ***p*<0.001

Table 34

*Standardised coefficients for each PID-5 domain from the excessive exercise behaviour model and age*

|  | Males | |  | Females | |
| --- | --- | --- | --- | --- | --- |
|  | Coefficient | *p* |  | Coefficient | *p* |
| Anhedonia | - | *-* |  | **0.33** | **<0.001** |
| Anxiousness | - | *-* |  | **0.18** | **0.002** |
| Deceitfulness | 0.00 | 0.991 |  | - | *-* |
| Distractibility | -0.01 | 0.878 |  | - | *-* |
| Eccentricity | - | *-* |  | 0.06 | 0.314 |
| Hostility | - | *-* |  | -0.01 | 0.850 |
| Manipulativeness | 0.01 | 0.880 |  | 0.05 | 0.314 |
| Perceptual Dysregulation | **0.21** | **0.027** |  | -0.01 | 0.796 |
| Rigid Perfectionism | - | *-* |  | 0.08 | 0.107 |
| Risk taking | **0.22** | **0.004** |  | - | *-* |
| Submissiveness | **0.22** | **0.008** |  | - | *-* |
| Unusual Beliefs and Experiences | 0.05 | 0.599 |  | - | *-* |
| Age | 0.03 | 0.685 |  | -0.02 | 0.626 |

Coefficient = Standardised regression weights**. Boldface** indicates a significant coefficient at the <0.05 level

# Muscle Building

## Step 2: Path model results for each PID-5 domain

Table 35

*Path model fit statistics for each PID-5 domain as predictors of muscle building behaviour*

|  | Males | | | |  | Females | | | |
| --- | --- | --- | --- | --- | --- | --- | --- | --- | --- |
|  | χ^2^ (*df*) | CFI | TLI | RMSEA  (90% CI) |  | χ^2^(*df*) | CFI | TLI | RMSEA  (90% CI) |
| Negative Affect | 12.40 (7) | 0.984 | 0.936 | 0.068  (0.000 – 0.129) |  | 2.77 (3) | 1.000 | 1.003 | 0.000  (0.000 – 0.083) |
| Detachment | 13.48**(1) | 0.967 | 0.504 | 0.274  (0.157 – 0.413) |  | 8.62*(1) | 0.991 | 0.869 | 0.139  (0.065 – 0.231) |
| Antagonism | 0.75 (1) | 1.000 | 1.016 | 0.000  (0.000 – 0.195) |  | 17.85**(1) | 0.973 | 0.592 | 0.207  (0.130 – 0.296) |
| Disinhibition | 2.50 (2) | 0.997 | 0.975 | 0.039  (0.000 – 0.164) |  | 5.34 (3) | 0.995 | 0.975 | 0.045  (0.000 – 0.105) |
| Psychoticism | 79.620**(1) | 0.316 | -3.104 | 0.688  (0.565 – 0.820) |  | 112.538**(2) | 0.710 | 0.129 | 0.375  (0.318 – 0.435) |

**p*<0.05, ***p*<0.001

Table 36

*Standardised coefficients for each PID-5 domain as a predictor of muscle building behaviour within domain path analyses for males and females*

|  |  | | | | Males | |  | Females | |
| --- | --- | --- | --- | --- | --- | --- | --- | --- | --- |
|  |  | | | | Coefficient | *p* |  | Coefficient | *p* |
| Negative Affect | | |  | |  |  |  |  |  |
|  | Emotional lability | | | | -0.07 | 0.549 |  | 0.06 | 0.459 |
|  | Anxiousness | | | | -0.17 | 0.087 |  | 0.01 | 0.942 |
|  | Separation Insecurity | | | | **0.18** | **0.030** |  | 0.06 | 0.343 |
|  | Submissiveness | | | | -0.07 | 0.384 |  | -0.02 | 0.783 |
|  | Hostility | | | | 0.08 | 0.379 |  | -0.02 | 0.770 |
|  | Perseveration | | | | 0.12 | 0.233 |  | **0.13** | **0.032** |
|  | Restricted Affectivity | | | | **0.21** | **0.004** |  | -0.04 | 0.486 |
| Detachment | | |  | |  |  |  |  |  |
|  | Withdrawal | | | | 0.05 | 0.624 |  | 0.03 | 0.656 |
|  | Intimacy avoidance | | | | -0.02 | 0.847 |  | -0.07 | 0.231 |
|  | Anhedonia | | | | -0.02 | 0.866 |  | -0.04 | 0.673 |
|  | Depressivity | | | | 0.00 | 0.998 |  | **0.21** | **0.025** |
|  | Suspiciousness | | | | 0.12 | 0.249 |  | 0.08 | 0.208 |
| Antagonism | | | |  |  |  |  |  |  |
|  | Manipulativeness | | | | 0.15 | 0.103 |  | 0.02 | 0.730 |
|  | Deceitfulness | | | | **-0.20** | **0.049** |  | 0.13 | 0.069 |
|  | Grandiosity | | | | 0.06 | 0.515 |  | 0.01 | 0.917 |
|  | Attention seeking | | | | 0.13 | 0.113 |  | 0.08 | 0.133 |
|  | Callousness | | | | **0.28** | **0.002** |  | -0.02 | 0.779 |
| Disinhibition | | |  | |  |  |  |  |  |
|  | Irresponsibility | | | | 0.14 | 0.094 |  | 0.03 | 0.590 |
|  | Impulsivity | | | | 0.09 | 0.356 |  | 0.07 | 0.330 |
|  | Distractibility | | | | **-0.20** | **0.031** |  | 0.07 | 0.204 |
|  | Risk taking | | | | **0.23** | **0.005** |  | 0.08 | 0.183 |
|  | Rigid perfectionism | | | | -0.05 | 0.490 |  | 0.06 | 0.216 |
| Psychoticism | |  | | |  |  |  |  |  |
|  | Unusual beliefs and experiences | | | | 0.11 | 0.173 |  | **0.14** | **0.032** |
|  | Eccentricity | | | | 0.04 | 0.648 |  | 0.08 | 0.148 |
|  | Cognitive and perceptual dysregulation | | | | -0.04 | 0.589 |  | 0.03 | 0.688 |

Coefficient = Standardised regression weights**. Boldface** indicates a significant coefficient at the <0.05 level

## Step 3: Results for composite path model

Table 37

*Path model fit statistics to predict muscle building behaviour in males and females*

|  | χ^2^(*df*) | CFI | TLI | RMSEA (90% CI) |
| --- | --- | --- | --- | --- |
| Males | 13.90 (9) | 0.979 | 0.918 | 0.057 (0.000- 0.113) |
| Females | 24.22**(1) | 0.910 | 0.097 | 0.243 (0.165 – 0.331) |

**p*<0.05, ***p*<0.001

Table 38

*Standardised coefficients for each PID-5 scale in the composite path model predicting muscle building behaviour for males and females*

|  | Males | | | |  | | Females | | | |  |
| --- | --- | --- | --- | --- | --- | --- | --- | --- | --- | --- | --- |
|  | Coefficient | | *p* | |  | | Coefficient | | *p* | |  |
| Anxiousness | | -0.09 | | 0.288 | |  | | - | | *-* | |
| Callousness | | **0.21** | | **0.014** | |  | | - | | *-* | |
| Deceitfulness | | -0.09 | | 0.266 | |  | | 0.10 | | 0.052 | |
| Depressivity | | - | | *-* | |  | | **0.13** | | **0.022** | |
| Distractibility | | **-0.18** | | **0.033** | |  | | - | | *-* | |
| Irresponsibility | | 0.12 | | 0.163 | |  | | - | | *-* | |
| Perseveration | | - | | *-* | |  | | 0.05 | | 0.424 | |
| Restricted Affectivity | | **0.16** | | **0.035** | |  | | - | | *-* | |
| Risk taking | | 0.14 | | 0.076 | |  | | - | | *-* | |
| Separation Insecurity | | 0.16 | | 0.050 | |  | | - | | *-* | |
| Unusual Beliefs and Experiences | | - | | *-* | |  | | 0.09 | | 0.079 | |

Coefficient = Standardised regression weights**. Boldface** indicates a significant coefficient at the <0.05 level

## Step 4: Composite path model predicting Body Dissatisfaction

Table 39

*Muscle building path model fit statistics to predict Body Dissatisfaction in males and females*

|  | χ^2^ (*df*) | CFI | TLI | RMSEA |
| --- | --- | --- | --- | --- |
| Males | 22.18 (16) | 0.976 | 0.933 | 0.048 (0.000 – 0.092) |
| Females | 7.47 (4) | 0.990 | 0.961 | 0.047 (0.000 – 0.099) |

**p*<0.05, ***p*<0.001

Table 40

*Standardised coefficients for each PID-5 scale from the muscle building behaviour model and age*

|  | Males | | | |  | | Females | | | |  |
| --- | --- | --- | --- | --- | --- | --- | --- | --- | --- | --- | --- |
|  | Coefficient | | *p* | |  | | Coefficient | | *p* | |  |
| Anxiousness | | **0.21** | | **0.010** | |  | | - | | *-* | |
| Callousness | | -0.08 | | 0.318 | |  | | - | | *-* | |
| Deceitfulness | | 0.01 | | 0.940 | |  | | **0.11** | | **0.026** | |
| Depressivity | | - | | *-* | |  | | **0.38** | | **<0.001** | |
| Distractibility | | 0.00 | | 0.984 | |  | | - | | *-* | |
| Irresponsibility | | **0.18** | | **0.025** | |  | | - | | *-* | |
| Perseveration | | - | | *-* | |  | | 0.10 | | 0.054 | |
| Restricted Affectivity | | -0.06 | | 0.410 | |  | | - | | *-* | |
| Risk taking | | **0.25** | | **0.001** | |  | | - | | *-* | |
| Separation Insecurity | | 0.13 | | 0.128 | |  | | - | | *-* | |
| Unusual Beliefs and Experiences | | - | | *-* | |  | | -0.01 | | 0.849 | |
| Age | | 0.02 | | 0.830 | |  | | -0.01 | | 0.783 | |

Coefficient = Standardised regression weights**. Boldface** indicates a significant coefficient at the <0.05 level
